# CMTT-JTracker: a fully test-time adaptive framework serving automated cell lineage construction

**DOI:** 10.1093/bib/bbae591

**Published:** 2024-11-17

**Authors:** Liuyin Chen, Sanyuan Fu, Zijun Zhang

**Affiliations:** Department of Data Science, College of Computing, City University of Hong Kong, Hong Kong SAR, China; Hefei National Laboratory for Physical Sciences at the Microscale and Department of Physics, University of Science and Technology of China, Hefei, Anhui, China; Department of Data Science, College of Computing, City University of Hong Kong, Hong Kong SAR, China

**Keywords:** cell segmentation, cell tracking, deep learning, test-time adaptation

## Abstract

Cell tracking is an essential function needed in automated cellular activity monitoring. In practice, processing methods striking a balance between computational efficiency and accuracy as well as demonstrating robust generalizability across diverse cell datasets are highly desired. This paper develops a central-metric fully test-time adaptive framework for cell tracking (CMTT-JTracker). Firstly, a CMTT mechanism is designed for the pre-segmentation of cell images, which enables extracting target information at different resolutions without additional training. Next, a multi-task learning network with the spatial attention scheme is developed to simultaneously realize detection and re-identification tasks based on features extracted by CMTT. Experimental results demonstrate that the CMTT-JTracker exhibits remarkable biological and tracking performance compared with benchmarking tracking methods. It achieves a multiple object tracking accuracy (MOTA) of $0.894$ on Fluo-N2DH-SIM+ and a MOTA of $0.850$ on PhC-C2DL-PSC. Experimental results further confirm that the CMTT applied solely as a segmentation unit outperforms the SOTA segmentation benchmarks on various datasets, particularly excelling in scenarios with dense cells. The Dice coefficients of the CMTT range from a high of $0.928$ to a low of $0.758$ across different datasets.

## Introduction

Cell behavior analysis plays a crucial role in biology and medicine, particularly in studying and quantifying various cell behaviors, such as migration, morphological variation, mitosis, collision, and apoptosis [1, 2]. However, traditional quantitative analysis often relies on manual intervention or parameter adjustment by well-trained professionals. In addition, traditional methods impose a significant computational burden and introduce complexity in identifying the optimal processing configuration. Considering the massive volume of cell image data obtained from current imaging technologies, the efficiency of the manual analysis is concerned. The automation of cell image analysis offers substantial improvements in data processing efficiency and the subjective biases of operators [3, 4].

**Figure 1:**
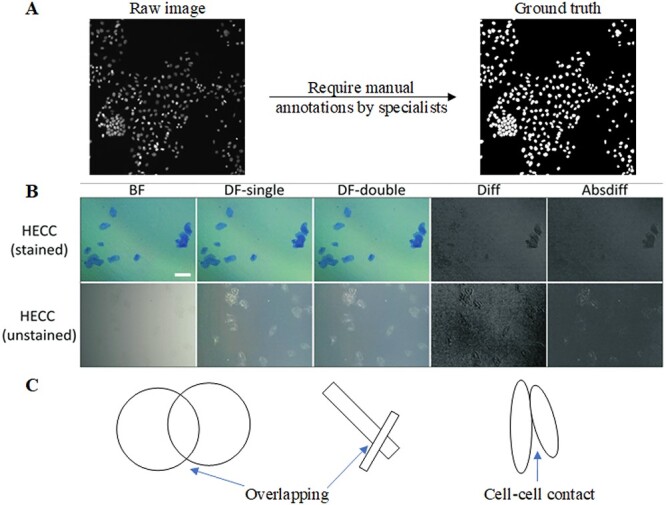
Challenges in processing cell images. (A) Annotation is tedious. (B) Subtracted images formed with single dark-field (DF-single) illumination pattern (second column) from images formed with double dark-field (DF-double) ring illumination pattern(third column) using regular arithmetic difference (Diff) or absolute difference (Absdiff), which resulted in the images shown in the fourth and fifth columns, respectively. [8] (C) Influence of cell overlapping.

Developing models for automating cell image processing faces three significant challenges. Firstly, a substantial training dataset is indispensable for capturing the variability in the features of the target cells. Since cell image labeling is tedious and expensive, the dataset may not contain a substantial amount of annotations (Fig. 1A). Secondly, it is imperative to address the disparities among various cell image datasets. These differences in distributions can be attributed to several factors, including variations in data collection settings, illumination, contrast, and the utilization of different image acquisition techniques. The choice of microscopy modality, such as fluorescence (Fluo), differential interference contrast (DIC), phase contrast (PhC), brightfield (BF), or darkfield (DF), can also contribute to these distribution discrepancies [5–7]. For instance, an illustrative example was provided in [8], involving human epithelial cheek cells captured under different settings and illumination patterns (Fig. 1B). Finally, the presence of a high cell density, overlapping between cells, or cell–cell contact poses challenges in cell segmentation (Fig. 1C). Microscopy data with these complexities often lead to inaccurate results. Overcoming these difficulties requires the development of more advanced algorithms.

Previous automated cell tracking methods can be broadly classified into two groups, tracking-by-model-evolution and tracking-by-detection. Tracking-by-model-evolution methods are unfortunately sensitive to overlapping, clustering, occlusion of cells, noise, and clutter. Moreover, tracking-by-detection algorithms generally demonstrate better tracking performance than tracking-by-model-evolution methods. Meanwhile, in tracking-by-detection algorithms, the segmentation units and trackers are typically independent, providing flexibility in further process optimization. These advantages have established tracking-by-detection methods as a popular direction in cell tracking research. However, tracking-by-detection methods also have notable limitations. Detailed discussions later will reveal instances where the biological performance of tracking-by-model-evolution methods can be more advantageous. Moreover, tracking-by-detection methods require significant computational resources and well-annotated data, which can hinder their application values.

In addition to the two common types of cell tracking methods mentioned above, in the field of multi-object tracking, the one-shot tracking method, the Joint Detection and Embedding (JDE) method [9], has been introduced. The vanilla JDE method employs a single network to generate the detection results and the corresponding appearance embeddings of the detected boxes simultaneously. In contrast, tracking-by-detection methods involve at least two computationally intensive components and two corresponding steps, a detector and a re-identification (ReID) model. Comparatively, the JDE method eliminates the need for re-computation and reduces computational requirements [9]. To further enhance tracking performance, various improved one-shot trackers incorporated more complex detection modules to achieve more impressive performance [10]. However, these complex detectors lead to increased computational complexity. Moreover, these methods overlook the inherent differences between detection and ReID [10], resulting in inferior performance compared with two-stage methods. Biologists often face limitations in computational resources compared with dedicated data scientists, yet they need to monitor a significant amount of cell images. Therefore, our objective is to develop a cell tracking method that minimizes the demand for computational resources while achieving performance comparable with two-stage tracking methods, such as the tracking-by-detection methods. Additionally, this method should effectively address the aforementioned challenges in cell image processing.

In the following sections, a review of existing cell segmentation and cell tracking methods will be provided. This will allow us to gain insight into the limitations of the current literature and identify the challenges that our proposed method aims to overcome.

### Existing cell segmentation works

Automated cell detection and segmentation pose significant challenges due to the complex patterns presented in cell image data including variations in modalities, stains, cell types, and cell densities [20]. In recent years, numerous attempts have been made to address the cell segmentation problem. Brief surveys summarizing the different approaches can be found in [21, 22]. These approaches can be broadly categorized into two groups, traditional methods and deep learning-based methods, which are briefly reviewed next. A summary of cell segmentation works reviewed in this study is offered in Table 1.

**Table 1:** Summary of existing cell segmentation works

Source	Segmentation methodology	Category	Evaluation
[11]	Intensity thresholding	Traditional-Unsupervised	Considered two BF datasets from **MuSCs** and **myoblasts** in **tracking**
[12]	Deformable model fitting	Traditional-Unsupervised	Considered wound healing images of **porcine epithelial** in preventing **cell merge events**
[13]	Morphological operations	Traditional-Unsupervised	Considered dermoscopy images, metrics applied include **Border Error, TDR and FPR**
[14]	Graph cut+watershed transform	Traditional-Unsupervised	Considered **Fluo** datasets, comparing with other methods
[15]	Semi-supervised hierarchical tree	Traditional-Supervised	Considered **three electron** microscopy datasets for 2D and 3D neuron segmentation
[16]	U-Net	Deep Learning	Considered archived thin blood smear images of human for **red blood cell detection**
[17]	U-Net	Deep Learning	Considered **CryoNuSeg** dataset for cell **nuclei** segmentation, metrics applied include **Dice, AJI** and **PQ**
[18]	Mask R-CNN	Deep Learning	Considered **two CTC** datasets with **DIC and PhC** microscopy images, metric applied is **IOU**
[19]	Mask R-CNN	Deep Learning	Considered **EVICAN** dataset for **cell** and **nucleus** segmentation, metric applied is **IOU**

**Traditional approaches:** Traditional unsupervised and supervised methods have both appeared in cell segmentation literature. The traditional unsupervised approaches for cell segmentation primarily relied on fundamental image processing techniques, such as the intensity thresholding [11], deformable model fitting [12], and morphological operations [13]. These methods could also serve as an initial step in the segmentation pipeline, providing a foundation for subsequent processing. For instance, in [14], graph cut segmentation was employed to segment individual cells, while the watershed transform was utilized to separate cell clusters.

Traditional supervised methods for cell segmentation, in contrast to unsupervised ones, incorporate manual feature selection. A key benefit of these methods lies in their high accuracy in segmenting specific cell types. Traditional supervised algorithms for cell segmentation [15] typically follow a structured process, which initially generates cell candidates, then derives hand-crafted feature representation, and finally employs classifiers using these features to identify target cell regions. However, the dependency on manual operations limits their flexibility and computing capability. Additionally, these methods often require extensive parameter tuning to enhance their performance.

**Deep learning-based methods:** The existing deep learning-based methods for cell segmentation mainly consider U-Net [23] or Mask R-CNN [24] as the backbone. In the literature, studies in [16, 17] discussed different variants of U-Net, and studies in [18, 19] developed various frameworks based on Mask R-CNN. U-Net is a simple and compact structure that can be easily modified and has a relatively low requirement for computing resources. Mask R-CNN is powerful in instance segmentation, easily outperforming all other methods on natural image datasets. However, both methods, even though they do not share the same principles, are supervised and rely on labeled data. Unlike natural image datasets, it is common in cell segmentation problems that the dataset is not well annotated. Cellular imaging can exhibit significant variability, even among identical cell types. The variations often arise from methodological differences in experimental execution or from variability among the researchers conducting the procedures. It is doubtful that supervised segmentation methods can maintain the required level of performance on these datasets with little or no annotated data. Therefore, in practical situations, it is preferred that the segmentation methods can be less dependent on data annotations and their quality.

### Existing cell tracking works

As listed in Table 2, previous cell tracking algorithms can be broadly classified into two main types, ”tracking-by-model-evolution” (also known as ”model evolution” or ”contour evolution”) algorithms and ”tracking-by-detection” algorithms. The former methods aim to address the segmentation and tracking tasks simultaneously [30–33]. They start with segmenting the cells in the first frame of a video and then evolve their contours by updating a probability map or energy function of cell regions in consecutive frames. These techniques rely on the crucial premise of clear spatiotemporal overlap among relevant cell regions. While effective for dispersed cells, these methods encounter difficulties in situations that cells are close with unclear boundaries or move over long distances [30]. The latter methods [11, 25, 26, 28, 29] aim to tackle the cell tracking problem with two modules, the segmentation unit and the tracker. Firstly, it involves the segmentation of cell outlines in all frames of a video. Subsequently, the detected outlines are linked together to form tracks. For the segmentation unit, traditional segmentation techniques [11, 28] or CNN-based methods [25, 26, 29] are commonly utilized. Trackers primarily aim to associate the detected cells between successive frames, typically relying on empirical association scores based on proximity and shape similarity [11, 28, 29]. Recent advancements in tracking methods [25, 26] have integrated inter-frame information to bolster associations. However, these methods often demand substantial training data for both detection and association, with certain techniques [25] struggling to perform effectively on small cells.

**Table 2:** Summary of existing cell tracking works

Source	Methodology	Category	Features	Evaluation
[25]	Recurrent hour-glass network	TBD	Considered extracting local features and memorizing inter-frame information; yet, performing poor on small cells	Considered CTC datasets for temporal or instance segmentation, combined instance segmentation and tracking
[26]	U-Net+Motion and Position Map (MPM) Tracker	TBD	Developed the cell motion field to represent cell association between successive frames	Considered public dataset [27] for cell detection, association accuracy and target effectiveness
[28, 11]	Intensity thresholding+Viterbi algorithm	TBD	Obtained tracks offering the largest possible increases to a designed scoring function	Considered 2 BF datasets with MuSCs and myoblasts for cell tracking
[29]	U-Net+PM Tracker	TBD	Considered the multi-frame input and the joint usage of 2 U-Nets, offering mitosis detection	Considered CTC datasets for cell tracking and detections
[30]	Deformable model fitting	TBME	Reformulated the deformable model in the discrete domain using 3D active meshes	Considered synthetic data and real Entamoeba histolytica parasites data for cell segmentation and tracking
[31]	Parameterization+Active contours	TBME	Developed the parametric model relying on exponential B-spline bases and a sphere-like topology	Considered both synthetic data and real olfactory glomeruli in mouse brain for cell segmentation and tracking
[32]	Gaussian Mixture-based model (GMM)	TBME	Applied Bayesian method with GMMs to recover cell lineages by connecting supervoxels spatially and temporally	Considered 3 model systems and 3 Fluo microscopy image types for segmentation and tracking
[33]	Graph cut+Chan–Vese model	TBME	Applied coherence-enhancing diffusion filtering and Chan–Vese model in the fast level set-like and graph cut frameworks	Considered 2D and 3D rat adipose mesenchymal stem cells and human lung squamous cell carcinoma cells

Note: TBD means tracking-by-detection, TBME means tracking-by-model-evolution in the table.

Tracking-by-model-evolution is fundamentally different from tracking-by-detection because it focuses on tracking mathematical representations of entire objects rather than just object locations. It is particularly appropriate for studying the morphological changes in cells observed at high magnification. However, tracking-by-model-evolution algorithms typically require a high imaging frequency to leverage temporal information and improve segmentation accuracy.

One challenge in tracking-by-model-evolution algorithms is initializing new cells that appear in the first image or migrate into the imaged area. This often requires a separate segmentation algorithm that works on a single image. Tracking-by-model-evolution algorithms evolve mathematical representations of cell contours by minimizing an energy function, which can be time-consuming [30, 33].

In contrast, tracking-by-detection algorithms can work with lower imaging frequencies. They have been widely adopted for studying cell migration and lineages at low magnification. These algorithms leverage temporal information to monitor cell movements and employ advanced data association techniques. One notable advantage of tracking-by-detection methods is their ability to address the tracking problem by solving segmentation and track-linking tasks separately. Thus, by replacing the segmentation algorithm, it is possible to apply a track-linking algorithm to new tracking scenarios [11]. The segmentation method developed in this work can also serve as a replacement.

In [22], 21 methods that perform well in the cell tracking challenge (CTC) [34] were compared using different metrics in 12 different datasets in CTC. Moreover, the segmentation strategy and tracking strategy considered by each method were listed in a categorized manner. From the comparison of these competing algorithms, it could be found that, in most practical scenarios, tracking-by-detection methods outperformed tracking-by-model evolution methods. However, a notable exception can be observed in datasets featuring high temporal resolutions with significant cell overlaps between frames. In such cases, tracking-by-model-evolution methods demonstrate superior capability in tracking cells over extended durations of videos compared with tracking-by-detection methods. Ulman *et al*. [22] suggested that, in specific scenarios, the biologically relevant performance of tracking-by-model-evolution methods surpasses that of the tracking-by-detection methods. This underscores the importance of accurately capturing complete and extended trajectories when assessing cell tracking methods. Such comprehensive long trajectories are essential in biologically representing the cyclical variations and movement patterns of the cells.

Indeed, in these situations, tracking-by-model-evolution methods seem to be more capable of tracking cells for longer stretches of the videos than tracking-by-detection methods. Paradoxically, this means that, even if the results of tracking-by-model-evolution methods are less similar to the ground truth solution, their biologically relevant performance might be higher in certain cases. Moreover, while tracking-by-model-evolution methods may be computationally intensive for tracking thousands of 3D objects, they offer intricate cell outlines, even amidst cell division [32].

Comparison of segmentation and tracking strategies in [22] confirms our argument for cell segmentation and tracking methods that, although previous deep learning-based methods often outperform traditional unsupervised methods, traditional unsupervised methods are still widely utilized to achieve a better generalization in the practical process. Furthermore, given that the cell image processing algorithm is primarily utilized by biologists, we should also consider making the algorithm implementation convenient. Such a consideration drives us to develop a fully test-time adaptation mechanism in cell image processing, which requires the trained model to be saved after the training and to be called directly by the algorithm in testing so that the requirement of further fine-tuning can be relieved.

On the other hand, the adoption of the JDE algorithm in cell tracking is not widespread. This approach aligns well with the concept of test-time adaptation, as it enables tracking within a single network and allows the reuse of network parameters. Therefore, we have specifically devised a JDE-based tracking algorithm with the test-time adaptation paradigm for pre-segmentation. This study aims to advance the performance of the proposed cell tracking algorithm to be better than that of the previous tracking methods on most datasets and to address certain shortcomings of the tracking-by-detection algorithm, i.e. the biological performance mentioned above.

### Proposed method CMTT-JTracker

**Figure 2:**
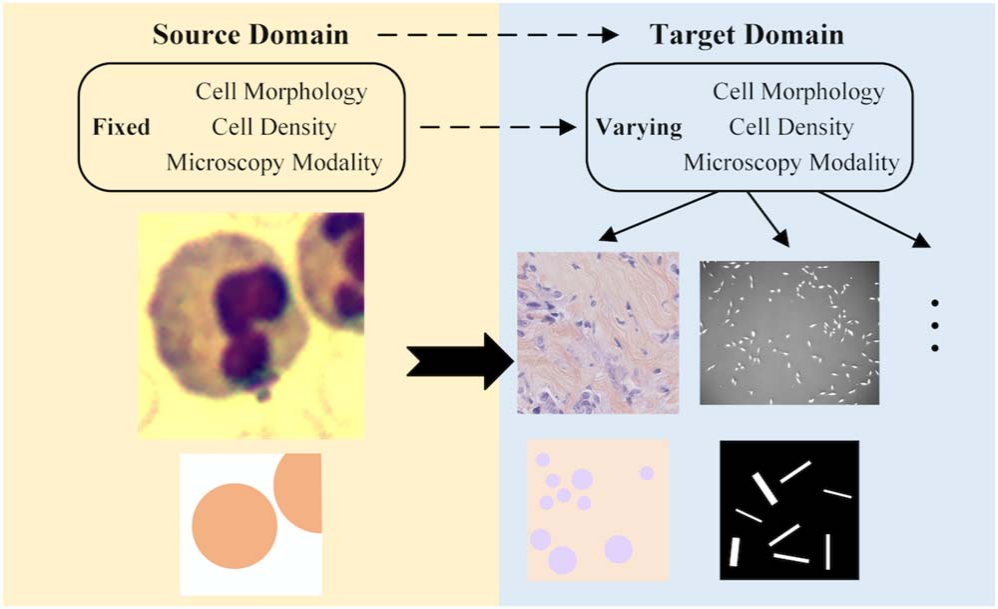
Sample images of previous cell datasets. (A1) BF-C2DL-HSC. (A2) BF-C2DL-MuSC. (A3) DIC-C2DH-HeLa. (A4) Fluo-C2DL-MSC. (A5) Fluo-N2DH-GOWT1. (A6) Fluo-N2DH-SIM+. (A7) Fluo-N2DL-HeLa. (A8) PhC-C2DH-U373. (A9) PhC-C2DL-PSC. (B1) BBBC003. (B2) BBBC004. (B3) BBBC005. (B4) BBBC006. (B5) BBBC007. (B6) BBBC008. (B7) BBBC009. (B8) BBBC010. (B9) BBBC018. (B10) BBBC019. (B11) BBBC020. (B12) BBBC030. (B13) BBBC039. (B14)-(B18) BBBC038. (C1) AndrewNuclei. (C2) CryoNuSeg. (C3) EVICAN. (C4) MoNuSeg. (C5) NIH-NLM-ThinBloodSmearsPf. (C6) PhaseContrast. (C7) segmentation-WBC. (C8) TNBC.

In this study, a central-metric fully test-time adaptive framework for cell tracking (CMTT-JTracker) is developed. Considering the aforementioned limitations of the previous tracking methods, our objective is to enhance the detection performance and generalizability of the tracking framework across diverse cell datasets. Meanwhile, it is imperative to effectively manage the training costs. Fully test-time adaptation methods allow for adaptation without relying on any data from the source domain or requiring additional training [35]. In the context of cell image processing, domain adaptation is illustrated in Fig. 2. To achieve this, we incorporate the test-time adaptation method into our framework, proposing a novel unsupervised two-stage testing paradigm called CMTT for cell pre-segmentation and detection processes. Furthermore, the simultaneous handling of detection and identification (ID) embedding tasks within a single network is regarded as a multi-task learning problem. Consequently, the feature maps processed by CMTT are directly forwarded to two separate task-specific branches, the detection branch and the ReID branch. Within the ReID branch, we construct a spatial attention ReID network (SA-ReID) to consolidate features from different scales and improve the learning of object-related representations. The main contributions of this study are summarized as follows:

- **From the application aspect:** A central-metric fully test-time adaptive tracking framework CMTT-JTracker is proposed to achieve stronger testing performance adaptiveness on cell tracking tasks. Extensive experimentation consistently demonstrates the superior accuracy and adaptiveness of our proposed CMTT-JTracker across multiple evaluation standards and various experimental settings using diverse datasets.
- **From the methodological aspect:** The proposed CMTT-JTracker possesses two advancements. First, in the CMTT part, a two-stage fully test-time adaptation mechanism for pre-segmentation is developed to improve the detection efficiency of the JDE tracking method. CMTT considers various cell types, imaging modalities, and cell densities, eliminating the need for additional training burdens. Meanwhile, a novel metric loss is specifically designed for patch adaptation in Stage 2 of CMTT. This patch adaptation stage incorporates microscopic features into the segmentation process. The second advancement is the spatial attention ReID network designed for enhancing the learning of object-related representations and consolidating features from multiple scales in the JDE. The effectiveness of the designed SA-ReID is well validated through ablation studies.
- **Data:** A unique dense cell image dataset with segmentation annotations named as SWARM is released in this work, which is the first time of such a kind of dataset in literature. Our proposed CMTT mechanism achieves a Dice score of $0.758$ on the SWARM dataset, surpassing all other benchmarks.

**Figure 3:**
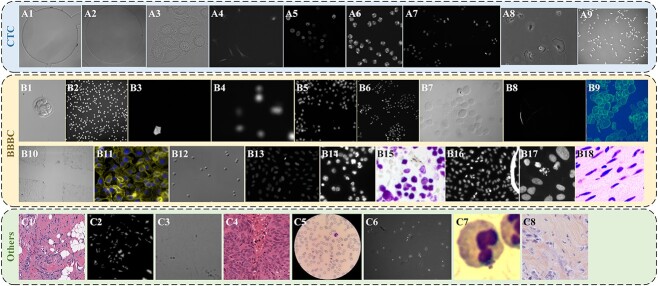
Domain adaptation in cell segmentation.

## Materials and methods

This section starts with a summary of publicly available cell image datasets with segmentation or tracking annotations. These efforts lay the foundation for the subsequent experimental validation of the cell lineage construction framework. Additionally, a novel cell dataset featuring segmentation annotations is introduced. Distinguished from the previous datasets, it boasts high cell density and considerable overlap. These features introduce greater complexity to the segmentation task and demand advanced capabilities from the segmentation models. Finally, details of our CMTT-JTracker method for cell tracking are provided.

### Summary of cell datasets with annotations

Large-scale databases possessing the cell segmentation or tracking annotation mainly cover the Broad Bioimage Benchmark Collection (BBBC) [36], Expert visual cell annotation (EVICAN) [19], and CTC [34]. In addition, there are several smaller scale cell image datasets that have been disclosed in previous studies [16, 27, 37–41]. They are described one by one next, and all sample images are provided in Fig. 3. Finally, we introduce our well-annotated cell segmentation dataset, SWARM.

**CTC:** The nine CTC datasets considered in this work are microscopy recordings of different live cells and organisms in two dimensions (2D). Sample images of these datasets are displayed in Fig. 2A1–A9. They also consist of different microscopy modalities including PhC, DIC, BF, and Fluo. They are summarized in Table 3. For more details, please refer to [22, 34]. These datasets are characterized by their ample data and uniformly formatted high-quality annotations. Additionally, [22] noted minimal cell overlap in CTC datasets. Given the previously discussed three challenges in cell segmentation, this implies that segmentation models for CTC datasets should focus predominantly on the variability of cell morphologies. Therefore, in order to test the adaptation performance of our framework, there is no need to test all CTC datasets, and a representative dataset of cells with different morphologies can be selected for validation.

**Table 3:** CTC 2D datasets summary

Dataset name	Cell sources	Cell imaging density	Image number	Image resolution	Contrast
BF-C2DL-HSC	Mouse hematopoietic stem cells in hydrogel microwells	1.8	3528 (57)	$1010\times 1010$	1.03
BF-C2DL-MuSC	Mouse muscle stem cells in hydrogel microwells	2.1	2752 (100)	$1070\times 1036$	1.01
DIC-C2DH-HeLa	HeLa cells on a flat glass	19.8	168 (18)	$512\times 512$	1.00
Fluo-C2DL-MSC	Rat mesenchymal stem cells on a flat polyacrylamide substrate	12.8	96 (51)	$992\times 832$	1.50
Fluo-N2DH-GOWT1	GFP-GOWT1 mouse stem cells	30.6	184 (50)	$1024\times 1024$	11.31
Fluo-N2DH-SIM+	Simulated nuclei of HL60 cells stained with Hoescht	58.2	215	$628\times 690$	1.23
Fluo-N2DL-HeLa	HeLa cells stably expressing H2b-GFP	15.8	184 (36)	$1100\times 700$	1.02
PhC-C2DH-U373	Glioblastoma-astrocytoma U373 cells on a polyacrylamide substrate	30.1	230 (34)	$696\times 520$	1.78
PhC-C2DL-PSC	Pancreatic stem cells on a polystyrene substrate	68.5	600 (4)	$720\times 576$	4.32

Note: The annotations for the simulated dataset are the exact reference annotations. The other datasets has computer-generated reference annotations, whose numbers are equal to the image numbers. The numbers of the human-made reference annotations are shown in the brackets of the image number.

CTC datasets utilize a variety of metrics, including internal cell signal heterogeneity and intercell signal variability, to characterize different subsets. However, many of these metrics are challenging to measure in other publicly available datasets, necessitating appropriate adjustments.

**Table 4:** BBBC 2D datasets summary.

Dataset name	Cell sources	Microscopy modality	Cell imaging density	Image number	Image resolution
BBBC003	Mouse embryos	DIC	2.2	15	$640\times 480$
BBBC004	Synthetic cells	Fluo	41.5	100	$950\times 950$
BBBC005	Synthetic cells	Fluo	3.1	19200	$696\times 520$
BBBC006	Human U2OS cells stained with Hoechst 33 342 markers	Fluo	10.7	52224	$696\times 520$
BBBC007	Drosophila melanogaster Kc167 cells	Fluo	67.2	32	$512\times 512$
BBBC008	Human HT29 colon-cancer cells stained with Hoechst and phalloidin	Fluo	71.3	24	$512\times 512$
BBBC009	Human red blood cells	DIC	50.2	5	$800\times 600$
BBBC010	C. elegans live/dead assay	BF and Fluo	3.3	200	$696\times 520$
BBBC018	Human HT29 colon-cancer cells stained with Hoechst 33342, pH3, and phalloidin	Fluo	80.2	168	$512\times 512$
BBBC019	Cell migration based on Melanoma cells, confluent DA3 cells, MDCK cells, confluent HEK293T cells and confluent MDCK/YFP-membrane cells	DIC	10.9	171	$1384\times 1028$
BBBC020	Bone-marrow derived macrophages from C57BL/6 mice stained with DAPI and CD11b/APC	Fluo	60.1	75	$1388\times 1040$
BBBC030	Chinese Hamster Ovary Cells	DIC	22.0	60	$1376\times 1032$
BBBC038	Cell nuclei derived from organisms including humans, mice, and flies	BF and Fluo	54.2	670	$937\times 546$
BBBC039	Nuclei of U2OS cells stained with Hoechst	Fluo	57.8	200	$520\times 696$

Note: If column ”Image number” contains parentheses, the number outside the parentheses represents the total number of cell images, while the number inside the parentheses represents the number of annotations. If no parentheses are included, the total number of cell images equals the number of annotations.

Table 3 illustrates the characteristics of the CTC datasets, utilizing the adjusted metrics. Five factors—cell sources, cell imaging density, image number, image resolution, and contrast—are included. The contrast ratio is retained in Table 3 to assess the differences at the pixel level between the cells and the background. Other statistics in [22] are omitted as they are not commonly available in public or lab datasets. Instead, cell sources are adopted to offer a simpler biological categorization. Cells from similar sources tend to have comparable characteristics, such as the shape and color. Additionally, we define cell imaging density, calculated as the effective area of the image divided by the cell count. Here, the ”effective area” refers to the region remaining after excluding marginal sections delineated by straight lines, which do not encompass intact cells. This factor simplifies automated computations by computer programs, compared with the cell density metric used in [22]. Meanwhile, it also offers an indication of the density of cells in the image. High cell imaging density signifies reduced intercellular spacing and raises the likelihood of overlapping cells and blurred boundaries. Consequently, it implies that the complexity of the segmentation task escalates. The volume of data is also crucial for effectively training cell segmentation algorithms; therefore, the numbers of images and the image resolutions in the datasets are enumerated to reflect this necessity.

**BBBC:** BBBC is a collection of freely downloadable microscopy image sets [36]. The summary of 2D datasets in BBBC is provided in Table 4. There are six types of annotations including counts, foreground/background, outlines of objects, biological labels, location, and bounding boxes. Each dataset can correspond to one or multiple annotations as ground truth. To simplify notation, foreground/background and outlines of objects annotations in this study are called as foreground and outlines annotations, respectively.

Datasets with foreground annotations can be directly applied to the benchmarking of a segmentation task, where the foreground file is the binary mask in the segmentation. Location and bounding box annotations include cell location information. They can be used as ground truth for cell detection but do not possess sufficient information for the cell boundary segmentation. The outlines of objects annotations include the specific boundary information of the cells, but cannot be used directly as a mask in the segmentation. Further data processing is thus required, i.e. the pixels inside of the border are filled with white for the cells, whereas the pixels outside of the border are filled with black for the background.

The BBBC currently consists of 54 datasets. Among them, nine are 2D datasets with foreground annotations, eight have outline annotations, and three have both annotations. Thus, a total of 14 datasets can be used as 2D segmentation benchmarks. The datasets containing foreground annotations are BBBC 003, 004, 005, 006, 008, 010, 019, 038, 039 [42]. The datasets containing outline annotations are BBBC 006, 007, 009, 010, 018, 020, 030 [43], 039. Sample images of these datasets except BBBC038 are shown in Fig. 2B1–B13. The images of BBBC038 come from a diverse collection of biological images (Fig. 2(B14–B18). The nuclei in BBBC038 are derived from a range of organisms including humans, mice, and flies. In addition, nuclei have been treated and imaged under a variety of conditions, including fluorescent and histology stains, several magnifications, and varying illumination quality.

**EVICAN:** EVICAN is a large-scale cell dataset, comprising partially annotated grayscale images of 30 different cell lines from multiple microscopes [19]. In general, there are 4640 partially annotated images and 98 fully annotated images. The sample image of EVICAN is shown in Fig. 2(C3).

**Other public cell segmentation datasets:** Seven additional publicly available datasets with cell segmentation annotations are reviewed in Table 5. Datasets (1)–(7) have been introduced separately in [16, 27, 37, 38, 39, 40], and [41]. These references offer the original papers or websites where the datasets are disclosed. To provide a comprehensive understanding of the datasets, additional details are included beyond the previously mentioned factors, such as cell sources, imaging density, image count, and resolution. The “Extra information” column specifies whether the dataset encompasses nucleus segmentation data, with “N/A” indicating its absence. In Table 5, “Image number” denotes the count of well-annotated images. The only exception is that, in the “Image number” column for PhaseContrast, “49 919” represents the total number of cell images, while “48” in parentheses indicates the quantity of images with high-quality annotation. Additionally, Fig. 2C(1–C8) displays sample images from these public datasets alongside EVICAN, aiming to minimize the image footprint.

**Table 5:** Public cell segmentation datasets summary

Dataset number	Dataset name	Cell source	Cell imaging density	Image number	Image resolution	Extra information
(1)	NIH-NLM-ThinBloodSmearsPf	Giemsa-stained thin-blood smear slides	44.5	165	$5312\times 2988$	N/A
(2)	PhaseContrast	C2C12 myoblast cell	23.1	49 919 (48)	$1392\times 1040$	N/A
(3)	segmentation-WBC	White blood cells	7.8	400	$120\times 120$ or $300\times 300$	Joint segmentation of cytoplasm and nuclei
(4)	CryoNuSeg	TCGA (H&E stainedWSI from human organs)	5.4	30	$512\times 512$	Nuclei segmentation
(5)	TNBC	H&E stained histology images from TNBC patients	14.2	50	$512\times 512$	Nuclei segmentation
(6)	MoNuSeg	TCGA (H&E stainedWSI from human organs)	59.2	51	$1000\times 1000$	Nuclei segmentation
(7)	AndrewNuclei	H&E stained ER+ breast cancer images	51.5	143	$2000\times 2000$	Nuclei segmentation

TCGA: The Cancer Genome Atlas; H&E: Hematoxylin and Eosin; TNBC: Triple Negative Breast Cancer; ER+: estrogen receptor positive Note: If column ”Image number” contains parentheses, the number outside the parentheses represents the total number of cell images, while the number inside the parentheses represents the number of annotations. If no parentheses are included, the total number of cell images equals the number of annotations.

**Our SWARM dataset:** We generated a dataset containing movies of bacteria swarming at the edge of a colony. The wild-type *Escherichia coli* K12 strain AW405 (HCB1) was used for swarm studies.

The swarm plates were prepared by following the protocol described previously. Swarm agar (0.45% Eiken agar, 1% Bacto peptone, 0.3% beef extract, and 0.5% NaCl) was poured to a polystyrene petri dish (150 mm diameter). Cells were first grown overnight in LB medium, subsequently diluted, and inoculated onto the surface of a swarm plate. They were then incubated at 30$^{\circ }$C and 100% relative humidity. This incubation continued until the swarming colony expanded to cover two-thirds of the swarm plate.

BF images of swarming bacteria were acquired with a Nikon Ni-E (Nikon, Minato City, Japan) upright microscope equipped with a sCMOS camera (C11440; Hamamatsu Photonics, Hamamatsu, Japan) at a frame rate of 50 fps with a 40 dry objective (Nikon CFI S Plan Fluor ELWD ADM 40XC, NA0.6, WD 2.8–3.6 mm). For the conditions of more sparse cells, a glass coverslip was placed at the swarm edge and the cells were imaged at 50 fps with a Nikon Apo $\lambda $ 60/1.40 NA oil-immersion objective.

**Figure 4:**
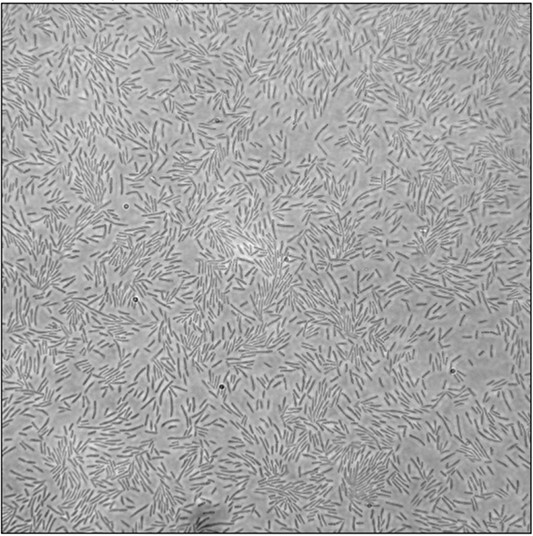
Sample image of the SWARM dataset.

This dataset contains a total of 20 023 images collected from eight different groups of bacteria, 50 of which have been well annotated by biologists. A sample image of the SWARM dataset is shown in Fig. 4. The cell imaging density is 155.8. Intuitively, the SWARM dataset comprises large image sizes with densely packed cells. The dataset will be publicly available after the manuscript is published.

### Overview of CMTT-JTracker

**Figure 5:**
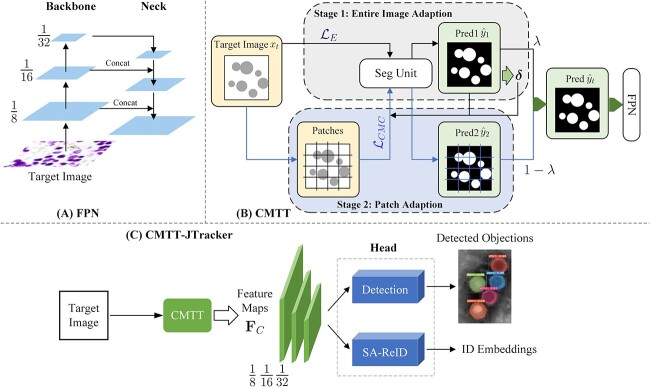
Architecture diagrams of CMTT-JTracker. (A) The network architecture of feature pyramid network (FPN). (B) The specific procedure of the unsupervised two-stage testing paradigm CMTT. (C) Illustration of the prediction procedure of CMTT-JTracker.

The general architecture diagrams of CMTT-JTracker are visualized in Fig. 5. Figure 5A depicts the network structure of FPN, while the unsupervised two-stage testing paradigm CMTT is described in Fig. 5B.

Figure 5C outlines the workflow of CMTT-JTracker. Firstly, to enhance feature representation learning for both detection and ReID tasks while reducing training complexity, a two-stage fully test-time adaptation mechanism CMTT is incorporated into CMTT-JTracker for the pre-segmentation of target images. The pre-segmentation step helps collect shared features between the detection and ReID tasks.

Next, addressing the detection and ID embedding tasks simultaneously within one network is treated as a multi-task learning problem. As a result, the feature maps $\mathbf{F}_{C}$ processed by CMTT are fed into two independent task-specific branches, the detection branch and the ReID branch.

**Figure 6:**
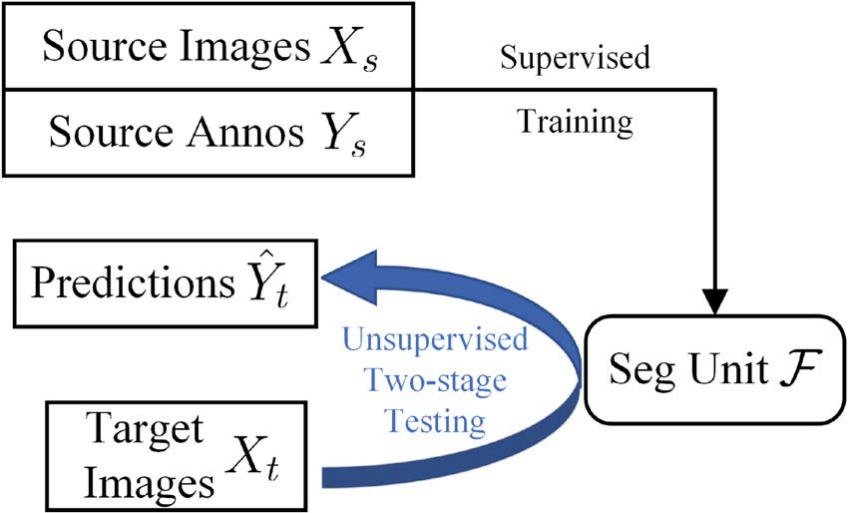
Illustration of the fully test-time adaptation procedure.

The detection branch consists of several stacked $1\times 1$ convolutional layers and produces the detection results. Non-maximum suppression [44] can be applied to these results to generate candidate boxes. On the other hand, the ReID branch utilizes a spatial attention ReID network, as illustrated in Fig. 6, to aggregate features from multiple scales and facilitate the learning of object-related representations.

A more comprehensive explanation of the CMTT mechanism is offered next, followed by a detailed introduction of the SA-ReID network architecture and the specific training details of CMTT-JTracker.

### CMTT paradigm

**Figure 7:**
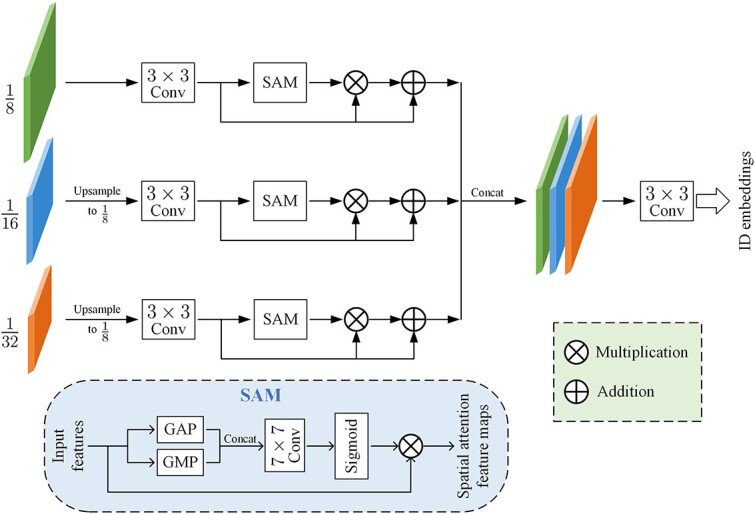
Illustration of the SA-ReID. SAM represents the SAM, Conv represents the convolutional layer, GAP represents the global average pooling layer, and GMP represents the global maximum pooling layer.

Figure 7 presents the overall flow of the fully test-time adaptation procedure. Let $(X_{s},Y_{s})=\{(x^{i}_{s},y^{i}_{s})\}_{i=1}^{N}$ denote the data and the corresponding labels of the source domain, and $X_{t} =\{x^{i}_{t}\}_{i=1}^{M}$ denote the data of the target domain. Firstly, the segmentation unit $\mathcal{F}$ is trained in the source domain using a soft dice loss. Subsequently, it undergoes adaptation to the target domain through an unsupervised two-stage testing process.

As shown in Fig. 5B, at Stage 1, the entire target images are taken as the adaptation objects and are input into the segmentation unit. A corresponding entire image loss $\mathcal{L}_{E}$ is applied to the trained segmentation unit for adaptation in this stage. The segmentation masks obtained from the adapted segmentation unit in Stage 1 are defined as prediction 1. The entire image adaptation and loss optimize the classification prediction for individual pixels in the entire image. However, they do not fully consider the situation in which the source domain and target domain differ significantly. If the cell imaging density in the target domain substantially exceeds that in the source domain, decision boundaries for pixel classification become challenging to identify. To resolve this, Stage 2 introduces patch adaptation. In Stage 2, the target images are split into patches first. Then these patches serve as the adaptation object with a central-metric contrastive (CMC) loss $\mathcal{L}_{CMC}$. This loss function is designed to facilitate differentiation of cells from the background, with the help of background information learned in Stage 1. Similarly, the segmentation masks obtained from the adapted segmentation unit in Stage 2 are defined as prediction 2. Comprehensive explanations of the loss functions, predictions, and $\delta $ for both stages will be detailed in the following section.

Finally, the predictions generated by the two stages are aggregated via weighted average to acquire the final prediction. Furthermore, for the next step of tracking, as well as yielding multi-resolution features, FPN is adopted as a feature extractor.

#### Objective functions

***Entire image loss: Shannon entropy and batch nuclear-norm loss***

First, our objective function considers minimizing the Shannon entropy [45]. Shannon entropy quantifies the uncertainty in model predictions. By reducing this uncertainty and aligning distributions across varying domains, it effectively improves model generalization [46]. This entropy is particularly advantageous in scenarios involving adaptation to new, unseen data during inference, aiding in the mitigation of domain shift effects [47, 48]. Suppose the soft label predicted by the trained model $\mathcal{F}$ of image $x_{t}$ is $\hat{y} = \mathcal{F}(x_{t})$, Shannon entropy is


(1) \begin{align*}& \mathcal{L}_{s} = H(\hat{y}) = -\Sigma_{c} p(\hat{y}_{c}) \log p(\hat{y}_{c})\end{align*}

For enhanced isolation of high-density cells, batch nuclear norm (BNM) loss [49] is further applied to classify each pixel in the entire image. BNM maximization has been proven to be capable of achieving better discriminability and diversity in label-insufficient learning situations, especially near the decision boundary. Suppose that the classification responses are obtained by the trained model $\mathcal{F}$, i.e. $A^{i} = \mathcal{F}(x^{i})$. The classification network consists of a feature extraction network, a classifier, and a softmax layer. Consider a batch randomly sampled with size $B_{t}$. This batch comprises samples denoted as $X_{t}^{B} = \{x_{t}^{i}\}_{i=1}^{B_{t}}$. The corresponding classification response matrix in the target domain $D_{t}$ is represented as $\mathcal{F}(X_{t}^{B})$. Following the notation in [49], the nuclear norm of a matrix $A$ is defined as $\Vert A \Vert _{*}$. Consequently, the loss function for BNM can be formulated as follows:


(2) \begin{align*}& \mathcal{L}_{bnm} = -\frac{1}{B_{t}} \Vert \mathcal{F}(X_{t}^{B}) \Vert_{*},\end{align*}

where the model $\mathcal{F}$ is trained in the source domain and then tested in the target domain. Minimizing $\mathcal{L}_{bnm}$ could reduce the data density near the decision boundary without losing diversity, which is more effective than typical entropy minimization. Meanwhile, the gradient of the nuclear norm could be calculated according to [50]. Thus, $\mathcal{L}_{bnm}$ could be applied to the gradient-based deep network training process.

For the adaptation of the entire image, Shannon entropy and BNM loss can be optimized simultaneously. Both loss functions can be combined through parameter $\alpha $:


(3) \begin{align*}& \mathcal{L}_{E} = \mathcal{L}_{s} + \alpha \mathcal{L}_{bnm}\end{align*}

***Central-metric contrastive loss***

There are three main challenges in patch segmentation. Firstly, when testing one of the patches individually, the overall information, such as position information and information about the neighboring patches, is unavailable. Secondly, if only the separately segmented patches are reassembled directly, it is likely that the edges will not be smooth. Thirdly, it is likely that the style of the patch and the full image will differ significantly, e.g. the full image may have more background information, but there may be mostly cell parts in one patch.

Many metric losses are proposed to simultaneously account for intra-class clustering as well as inter-class separation among the categories [51]. The CMC loss is proposed to take advantage of the overall information contained in a complete image to extract similar and different samples in patch segmentation, thus overcoming the previously mentioned challenges. The core idea of this loss is to obtain the typical information of the cell and background as the center, and then extend it outwards to obtain the complete segmentation. The process is to take the pixel information of the cell and background learned from the corresponding region of the patch in Stage 1 as the center $\delta $, and compare the similarity of the pixels in the patch with this information.

We denote a certain patch as $P$, $\delta $ as the median or mean of the pixels in the background area predicted in Stage 1, the set of background pixels obtained by Stage 1 in the patch as $P^{-}$, and the set of cell pixels as $P^{+}$. Empirically, normalized inverse Euclidean distance [52] $\phi $ is taken as the similarity metric between pixels $i$ and $j$:


(4) \begin{align*}& \phi (i, j) = \frac{1}{1 + \Vert i - j \Vert^{2}}.\end{align*}

This information is then utilized to pull similar samples across sources and targets closer to each other while pushing away dissimilar samples, with our CMC loss given by


(5) \begin{align*}& \mathcal{L}_{CMC} = -\log \frac{\Sigma_{i\in P^{+}} e^{\phi(i, \delta)}}{\Sigma_{i\in P^{+}} e^{\phi(i, \delta)} + \Sigma_{i\in P^{-}} e^{\phi(i, \delta)}}\end{align*}

Contrastive loss is shown to work well for large intra-class variations empirically in [53, 54] and theoretically in [55]. CMC loss demonstrates similar benefits while additionally accounting for possible domain gaps between the background and cell pixels.

#### Modulation parameters

Since only the target data are available in the test, modifying all the parameters of the model is unstable and inefficient [46]. Instead, we only update the parameters in the batch normalization (BN) layers for the fully test-time adaptation. According to [56], the normalization statistics for each domain are different; if convolutional layers learn the knowledge of feature extraction, then the BN layers are the key to the domain shift. Furthermore, BN layers have much fewer parameters to optimize, leading to faster adaptation. Therefore, during test-time optimization, the model parameters are frozen, except those of batch norm layers. Let $X \in RB\times P$ denote the input to the BN layer, where $B$ denotes the batch size and $P$ is the feature dimension. For the feature $j \in \{1, \cdots , P \}$, the BN layer first centers and standardizes $x_{j}$ into $\hat{x}_{j}$ and then transforms it by affine parameters $\gamma $ and $\beta $.


(6) \begin{align*}& \hat{x}_{j} = \frac{x_{j} - E[X_{j}]}{\sqrt{Var[X_{j}]}},\quad y_{j}= \gamma_{j} \hat{x}_{j} + \beta_{j}\end{align*}

For the test-time optimization, the mean and standard deviation are estimated from the target data, while the affine parameters $\gamma $ and $\beta $ are optimized by the gradient back-propagation from the loss function.

### Spatial attention ReID network

A spatial attention ReID network is proposed in this section, as shown in Fig. 7. It is designed to aggregate features from different resolutions and ensure semantic alignment of ID embeddings for target cells of varying scales. Our ReID branch draws inspiration from [10]. However, considering that the preceding CMTT already handles cells of different resolutions and scales differently, we simplify the ReID model from [10] to reduce complexity and computational requirements. Specifically, SA-ReID does not further extract information from different resolutions but instead fuses this information, aiming to focus the model’s attention on the object-related features.

Spatial attention plays a crucial role in capturing and highlighting spatially significant regions within an image or feature map [57]. The spatial attention module (SAM) module employs average pooling and max pooling operations on the channel dimension of the input features, generating two 2D maps. These maps are then concatenated and sequentially processed through a $7\times 7$ convolutional layer and a sigmoid layer, obtaining a feature map. For each resolution, the feature map is learned individually and then fused with the original feature using element-wise multiplication, yielding a spatial attention map.

In SA-ReID, firstly, the features from the $\frac{1}{16}$ and $\frac{1}{32}$ scales (compared with the input image size) are upsampled to $\frac{1}{8}$. Subsequently, a $3\times 3$ convolutional layer is applied to encode the upsampled feature maps. To better capture useful information for each target at different resolutions, the SAM is incorporated after the convolutional layer. This module enhances target-related features while suppressing background noise. The spatial feature map generated by SAM is then aggregated with the feature map before SAM using multiplication and addition operations. Furthermore, the feature maps from different scales are concatenated. Finally, a $3\times 3$ convolutional layer is employed to map the features to the corresponding ID embeddings. Each ID embedding represents the identity information of the object at location $(x, y)$, which can be extracted based on the detection results for the subsequent ReID task.

### Training details

For jointly optimizing the object detection and ReID tasks, a weighted sum of detection losses and ReID losses should be minimized. Here, the detection losses consist of classification loss and box regression loss. Specifically, the ground-truth annotation for an input image is denoted as $\{B_{i}\}$, where $B_{i} = (x^{(i)}, y^{(i)}, w^{(i)}, h^{(i)}, c^{(i)})$. $(x^{(i)}, y^{(i)})$ indicate the coordinates of the center of the bounding box and $(w^{(i)}, h^{(i))}$ indicate the size of the bounding box. $c^{(i)}$ is the ID index that the object belongs to the $i$th cell. $C$ is the number of ID in the training dataset, which denotes the number of cells. For each location $(x, y)$ on the detection result maps of different resolutions, it is denoted as a 5D vector $t = (x^{*}, y^{*}, w^{*}, h^{*}, p)$, following the standard anchor-based protocol [58]. Here $(x^{*}, y^{*}, w^{*}, h^{*})$ are the bounding box predictions and $p$indicates the foreground probability of the bounding box. For foreground and background classification, we define the anchor points in the location $(x, y) = ( \lfloor \frac{x^{(i)}}{r} \rfloor , \lfloor \frac{y^{(i)}}{r} \rfloor )$ as positive samples ($r$ is the downsample ratio, i.e. 8, 16, 32, and $\lfloor \cdot \rfloor $ indicates rounding down to an integer). Given that the morphological and density variations that may exist in cell detection problems, focal loss [59] is utilized as the classification loss to address class imbalance issues and emphasize challenging samples. It can be formulated as


(7) \begin{align*}& \mathcal{L}_{cls}(p_{t}) = -\alpha_{c} (1 - p_{t})^\gamma_{c} \log (p_{t}),\end{align*}

where


(8) \begin{align*}& p_{t} = \begin{cases} p & if (x, y) = \left( \left\lfloor \frac{x^{(i)}}{r} \right\rfloor, \left\lfloor \frac{y^{(i)}}{r} \right\rfloor \right), \\ 1 - p & otherwise\\ \end{cases}\end{align*}

Following [59], the parameters are set as $\alpha _{c} = 0.25$ and $\gamma _{c} = 2$.

For faster convergence and better performance, CIOU loss [60] is employed as the box regression loss, which can be defined as


(9) \begin{align*}& \mathcal{L}_{reg}(\hat{b}_{x, y}) = \begin{cases} 1 - \mathcal{C}(b_{i}, \hat{b}_{x, y}) & if (x, y) = \left( \left\lfloor \frac{x^{(i)}}{r} \right\rfloor, \left\lfloor \frac{y^{(i)}}{r} \right\rfloor\right ), \\ 0 & otherwise\\ \end{cases}\end{align*}

where $\mathcal{C}$ represents the CIOU operation. $b_{i}$ indicates the ground-truth box and $\hat{b}_{x, y}$ is the box prediction at location $(x, y)$ of feature maps. We define our detection loss as follows:


(10) \begin{align*}& \mathcal{L}_{det} = \frac{1}{N_{pos}} \sum_{i}^{M} \sum_{x, y} \left(\mathcal{L}_{cls}\left(p_{x, y}^{i}\right) + \beta_{c} \mathcal{L}_{reg}\left(\hat{b}_{x, y}^{i}\right)\right),\end{align*}

where $M$ is the number of the resolutions and $N_{pos}$ denotes the number of positive samples. $\beta _{c}$ indicates the loss weight. We set $\beta _{c} = 0.05$ as that in YOLO-v5.

The definition and training of ReID loss follow the vanilla JDE tracker[9], which uses the cross-entropy loss as the objective for ID embedding learning. In this work, we model this task as a classification task, and use a fully connected layer to map ID embeddings to a class distribution vector $P = \{p(c), c \in [1, 2, \cdots , C]\}$. By comparing with the one-hot representation of the ground-truth class label $Y^{i}(c) \in \mathbb{R}^{C\times 1\times 1}$, the ReID loss can be computed as


(11) \begin{align*}& \mathcal{L}_{id} = \frac{1}{N} \sum_{i=1}^{N} \sum_{c=1}^{C} Y^{i}(c) \log (p(c)),\end{align*}

where $N$ denotes the number of objects in the current image.

The joint loss can be written as a weighted linear sum of detection loss $\mathcal{L}_{det}$ and ReID loss $\mathcal{L}_{id}$, as


(12) \begin{align*}& \mathcal{L}_{total} = \mathcal{L}_{det} + \eta \mathcal{L}_{id},\end{align*}

where $\eta $ is set to 0.05 to balance the objective detection and ReID tasks as justified by the preliminary experiments offered in Supplementary File Section 2.

The main focus of this paper does not lie in the online association algorithm. Thus, we maintain the original settings of the JDE framework [9].

## Experimental results

### Implementation details and benchmarking methods

In the experiment, the state-of-the-art architecture U-Net++ [61] is adopted as the segmentation unit in CMTT. For all the experiments, the Adam optimizer is used for 100 epochs with a learning rate equal to 0.0001. $\alpha $ is set to $0.5$ and the proportion of the dataset to include in the train split is set to $60\%$ unless otherwise specified. No data augmentation method is adopted during training and testing. Training of the U-Net++ model in the source domain (100 epochs) takes $\sim $1 h and the adaptation in the target domain (<10 epochs) takes ¡5 min on an NVIDIA GPU 3080Ti. Two popular tracking-by-detection methods are introduced as benchmarks.

1. KTH Tracker [28]: This tracker has been reported to perform best in the CTC [22].
2. Bayesian Tracker (BTracker) [62]: This tracker is designed for multi-object tracking, widely used to reconstruct trajectories in crowded fields.
3. MPM Tracker [26]: This tracker jointly represents both detection and association to ensure coherence. It is a simple but powerful method for multi-object tracking in dense environments and outperforms the state-of-the-art methods significantly.

Gaussian Mixture-based Tracker (GMM) [32], a typical tracking-by-model-evolution method, is also adopted as the benchmarking method. Moreover, the vanilla JDE method in [10] is employed for comparison.

In addition to performing experiments using CMTT-JTraker as a tracking method on the datasets, we also employ CMTT as the segmentation unit in the tracking-by-detection methods. In the tracking-by-detection approaches, our CMTT and U-Net can replace the segmentation units in existing benchmarks. This allows us to assess the impact of different segmentation methods on both tracking outcomes and biological performances.

All the benchmarks are publicly available. The parameters are provided in the software package and then optimized for each dataset.

### Evaluation metrics

Multiple object tracking accuracy (MOTA)[63] is taken as a global measure to evaluate the overall tracking performance.

To demonstrate the biological properties of the different methods, complete tracks (CTs) and track fraction averages (TFs) are defined based on [22]. CT represents the proportion of cell tracks that can be fully reconstructed by the given method relative to the ground truth tracks. CT measures the ability of a method to reconstruct complete cell lineages. TF is the fraction of the longest continuously matching algorithm-generated tracklet among all detected tracks, with respect to the reference track. Simply speaking, TF is the proportion of ground truth trajectories that can be accurately identified, given that only those cells that can be detected by the segmentation algorithm are considered.

### Performance comparison on CTC datasets

The results in Table 6 show that our CMTT-JTracker outperforms all other benchmarking methods in different datasets on various metrics. Moreover, when our CMTT is considered as the segmentation unit instead of U-Net, BTracker, KTH Tracker, and MPM Tracker all exhibit enhanced tracking performance. On the other hand, GMM, as the tracking-by-model-evolution method, offers a significantly lower MOTA. However, on the dataset Fluo-N2DH-SIM+, the biological performance of the tracking-by-model-evolution method is better than the benchmarking tracking-by-detection methods, i.e. the CT and TF values are higher. This finding is also consistent with the findings offered in [22]. The biological performance of our method is also significantly better than that of U-Net.

**Table 6:** Tracking results on CTC datasets (Fluo-N2DH-SIM+ and PhC-C2DL-PSC)

		Fluo-N2DH-SIM+			PhC-C2DL-PSC		
		MOTA$\uparrow $	CT$\uparrow $	TF$\uparrow $	MOTA$\uparrow $	CT$\uparrow $	TF$\uparrow $
BTracker	U-Net	0.760	0.245	0.712	0.812	0.180	0.854
	CMTT	0.854	0.461	0.899	0.839	0.309	0.854
KTH Tracker	U-Net	0.781	0.309	0.804	0.833	0.247	0.809
	CMTT	0.878	0.471	0.908	0.842	0.340	0.872
MPM Tracker	U-Net	0.802	0.335	0.837	0.838	0.270	0.826
	CMTT	0.885	0.486	0.919	0.845	0.347	0.877
GMM		0.792	0.403	0.884	0.801	0.134	0.793
vanilla JDE		0.822	0.452	0.808	0.821	0.197	0.823
CMTT-JTracker		0.894	0.499	0.932	0.850	0.373	0.884

**Figure 8:**
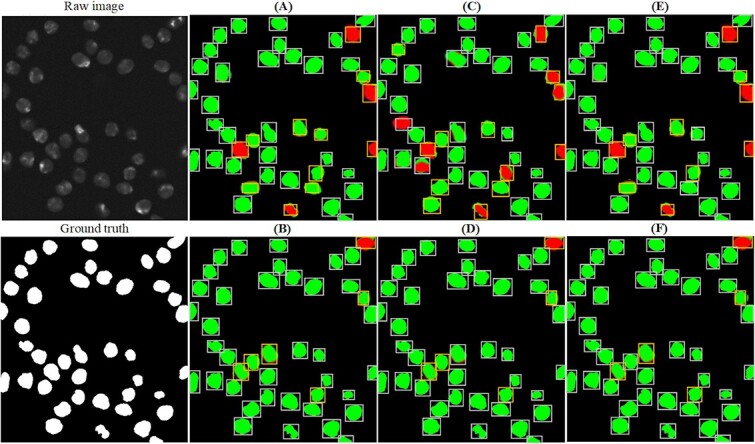
Detection results on Fluo-N2DH-SIM+ when using different tracking methods. (A) U-Net + KTH Tracker, (B) CMTT + KTH Tracker, (C) vanilla JDE, (D) CMTT-JTracker, (E)U-Net + MPM Tracker, (F) CMTT + MPM Tracker.

Figure 8 presents a comparison of detection results between our CMTT-JTracker and three benchmarking methods. The correct segmenting results are represented by the green color, while the red color indicates incorrect segmenting results. Accurate detection results are denoted by white boxes, while the yellow boxes represent significant deviations from the ground truth. It can be observed that the vanilla JDE exhibits the poorest performance in terms of segmentation and detection, primarily due to the competition between the detection and ReID tasks in the one-shot method [10]. The performance of U-Net surpasses that of vanilla JDE but falls short of our approach. Regarding CMTT+KTH Tracker, CMTT+MPM Tracker, and CMTT-JTracker, there are minimal differences in segmentation results, but their detection results vary. This discrepancy arises from the fact that CMTT+KTH Tracker and CMTT+MPM Tracker handle the detection and ReID tasks independently in two separate stages, while CMTT-JTracker is a one-shot method. While CMTT+KTH Tracker and CMTT+MPM Tracker successfully avoid the competition between the detection and ReID tasks, they also sacrifice the ability to leverage the shared knowledge between these two tasks. Based on the comparisons in Fig. 8 and Table 6, it is evident that CMTT-JTracker outperforms CMTT+KTH Tracker and CMTT+MPM Tracker, validating that our design mitigates the performance degradation caused by competition. Moreover, the overall performance of the MPM Tracker surpasses that of the KTH Tracker and BTracker. Additionally, due to its enhanced utilization of inter-frame information, it exhibits superior performance in challenging scenarios, such as dense cell populations and long cell trajectory movements.

**Figure 9:**
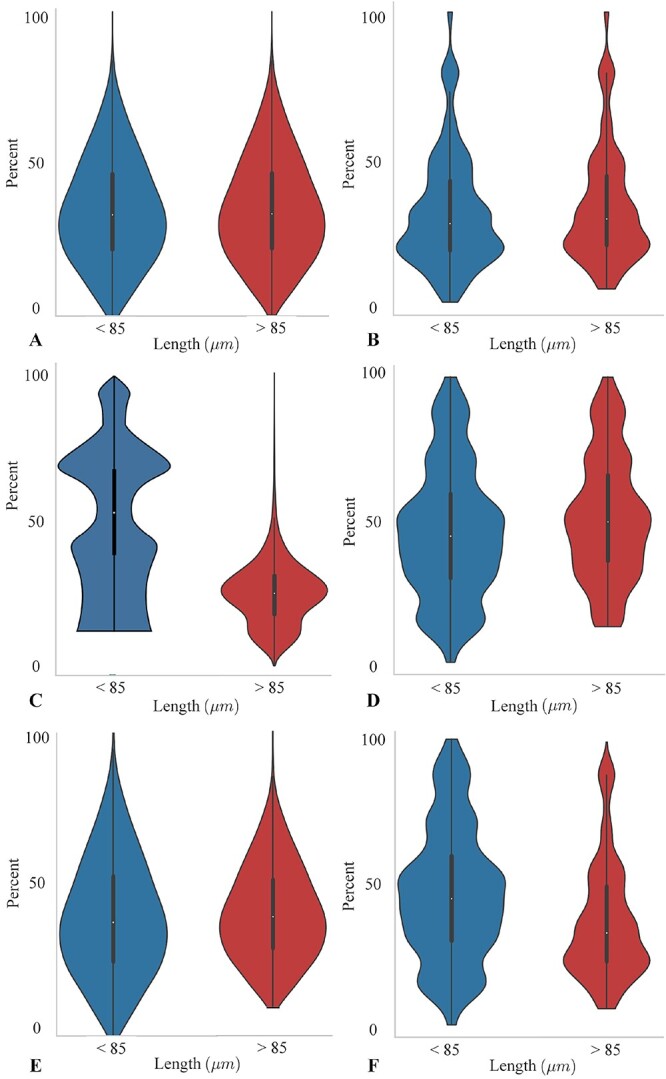
Violin plots for performances of different models. The vertical axis of the violin plot represents the percentage of correctly predicted trajectory lengths relative to the ground truth trajectory lengths, while the horizontal axis denotes the lengths of the predicted trajectories, regardless of their correctness. (A) U-Net + KTH Tracker, (B) CMTT + KTH Tracker, (C) vanilla JDE, (D) CMTT-JTracker, (E)U-Net + MPM Tracker, (F) CMTT + MPM Tracker.

**Figure 10:**
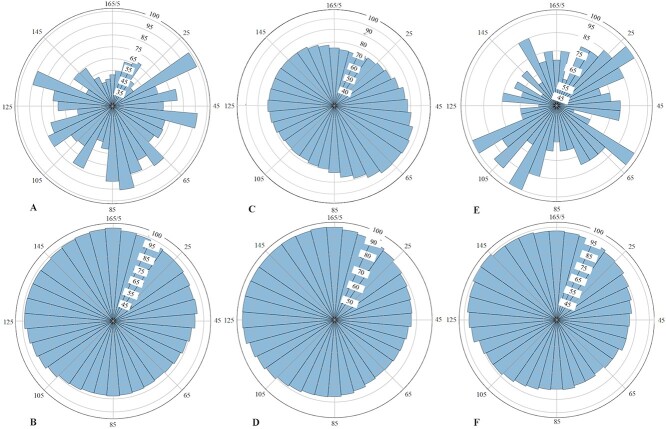
Angle histograms for performances of different models. In these histograms, the angles correspond to different ground truth trajectory lengths, while the polar coordinates indicate the percentage of correctly predicted trajectories among all trajectories of corresponding lengths in the ground truth dataset. (A) U-Net + KTH Tracker, (B) CMTT + KTH Tracker, (C) vanilla JDE, (D) CMTT-JTracker, (E)U-Net + MPM Tracker, (F) CMTT + MPM Tracker.

Acknowledging the significance of complete and extended trajectories in facilitating the exploration of cellular activities by biologists, the inclusion of comprehensive trajectory data has been prioritized. To provide additional insight into the relationship between different tracking methods and trajectory length, we have generated violin plots (Fig. 9) and angle histograms (Fig. 10) illustrating the performances of the models in the Fluo-N2DH-SIM+ dataset. Figures 9 and 10 reveal that the vanilla JDE method exhibits relatively better performance in trajectories of moderate length. The relatively poorer performance of the vanilla JDE method in longer trajectories can be attributed to its lack of explicit consideration for trajectory continuity. This deficiency hampers its effectiveness in handling longer trajectories. Furthermore, the presence of shorter trajectories poses challenges in the competition between the detection and ReID tasks. As mentioned in [10], the competition between tasks can potentially lead to ambiguous learning, wherein the pursuit of high performance in one task may result in performance degradation or stagnation in the other task. In contrast, the performance of U-Net does not show a clear correlation with trajectory length. However, our approaches, which take into account both biological performance and trajectory coherence, demonstrate superior performance in longer trajectories and also achieve commendable results in medium and short trajectories. Notably, the overall performance of CMTT-JTracker exceeds those of CMTT+KTH Tracker and CMTT+MPM Tracker. The performance of the MPM Tracker, illustrated in Figs 9 and 10, exhibits a comparable trend with the KTH Tracker regarding trajectory length variability. Nonetheless, the MPM Tracker performs better than the KTH Tracker especially in long-trajectory scenarios.

To demonstrate the stability of CMTT-JTracker, we conduct four-fold cross-validation (CV) on the Fluo-N2DH-SIM+ dataset and validate the framework on the independent Fluo-N2DL-HeLa dataset after training on the Fluo-N2DH-SIM+ dataset. The experimental results are presented in Table 7. For comparison purposes, the MPM Tracker, highlighted as the top performer in Table 6, also undergoes cross-validation and independent validation. During the four-fold CV, the low standard deviations of MOTA, CT, and TF signify the stability of CMTT-JTracker. Additionally, the consistency between the CV and the independent validation results further exhibits the robustness of CMTT-JTracker.

**Table 7:** Performance comparison on Fluo-N2DH-SIM+ with four-fold CV (mean$\pm $std) and independent validation on Fluo-N2DL-HeLa

		4-fold CV			Fluo-N2DL-HeLa		
		MOTA$\uparrow $	CT$\uparrow $	TF$\uparrow $	MOTA$\uparrow $	CT$\uparrow $	TF$\uparrow $
MPM Tracker	U-Net	0.809$\pm $0.024	0.337$\pm $0.038	0.842$\pm $0.030	0.753	0.230	0.798
	CMTT	0.887$\pm $0.011	0.489$\pm $0.014	0.918$\pm $0.012	0.876	0.442	0.894
CMTT-JTracker		0.899$\pm $0.009	0.505$\pm $0.005	0.933$\pm $0.006	0.892	0.478	0.914

**Figure 11:**
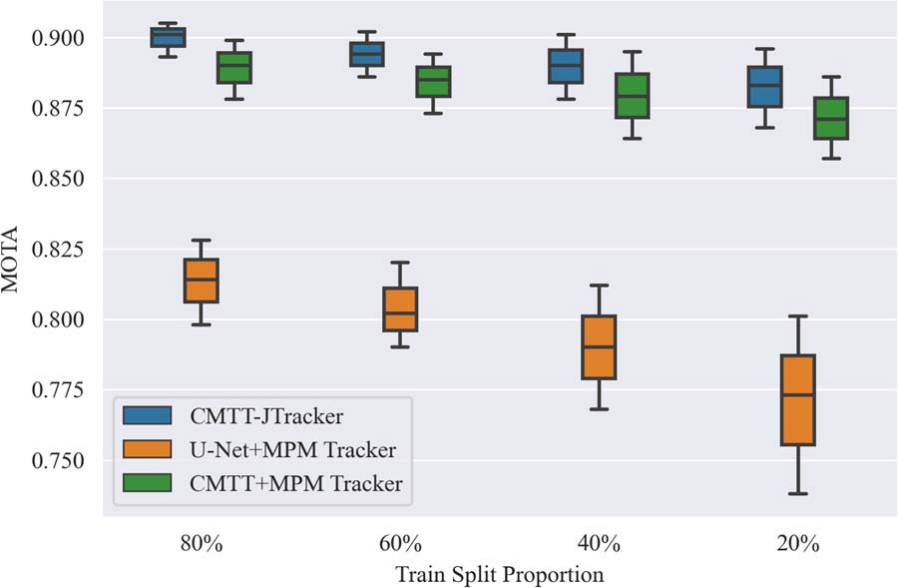
Model performance changes with different train split proportions on Fluo-N2DH-SIM+.

Moreover, the stability of CMTT-JTracker is evaluated by comparing it with the previously top-performing baseline methods through varying the training and validation split ratios within the Fluo-N2DH-SIM+ dataset. The performances of these models versus the train split proportion are shown in Fig. 11. It is evident that CMTT-JTracker surpasses all other methods in terms of MOTA. Additionally, it can be concluded that CMTT-JTracker consistently delivers the best performance in all training ratios. The model’s advantage becomes more pronounced with an increase in training samples, as indicated by the performance trend relative to the train split proportion.

### Ablation studies

Comprehensive ablation studies were conducted to assess the impact of different modules within the proposed network. This section focuses on the contributions and performance enhancement of two modules, CMTT and SA-ReID, in the tracking performance of the entire framework. In the ablation experiments, these two modules were individually replaced with the baseline methods to ensure the normal functioning of the JDE framework. Specifically, CMTT was substituted with FPN, and SA-ReID was replaced with stacked convolutional layers. The overall tracking performance was evaluated using MOTA, while CT was adopted to measure the completeness of the predicted trajectories compared with the ground truth. Additionally, to gain further insight into the ReID performance, two additional metrics, MT and ML, were reported [10]. The Most Tracked ratio (MT) denotes the percentage of tracked targets that achieve a successful match with the Ground Truth for at least 80% of the time. It indicates the proportion of tracked objects that consistently maintain a high level of accuracy in their tracking performance. Conversely, the Most Lost ratio (ML) represents the percentage of tracked targets that exhibit a successful match with the Ground Truth for ¡20% of the time. It signifies the proportion of tracked objects that experience a low level of accuracy in their tracking performance, with a significant portion of their tracking instances failing to align with the ground truth.

Table 8 presents the performance of various tracking frameworks with different compositions across these metrics. The performance of the SA-ReID branch surpasses that of the convolutional layers in terms of MT and ML. This aligns with our design intention to enhance attention toward the targets. The inclusion of CMTT as a fundamental segmentation module for tracking contributes to the framework’s overall performance improvement across different metrics. Notably, when designing SA-ReID, we took into consideration that CMTT had already extracted information from features of different resolutions. In SA-ReID, we further aggregated this information, resulting in a significant enhancement when both CMTT and SA-ReID are used together, surpassing their individual performances.

**Table 8:** Ablation studies of the proposed CMTT-JTracker on Fluo-N2DH-SIM+ dataset

Modules				MOTA$\uparrow $	CT$\uparrow $	MT$\uparrow $	ML$\downarrow $
FPN	CMTT	Conv	SA-ReID				
✓		✓		0.834	0.465	15.1	24.1
	✓	✓		0.855	0.476	24.5	20.9
✓			✓	0.861	0.484	26.9	20.5
	✓		✓	$\boldsymbol{0.894}$	$\boldsymbol{0.499}$	$\boldsymbol{29.3}$	$\boldsymbol{16.4}$

Furthermore, it is worth noting that CMTT can be independently utilized as an unsupervised segmentation module. The remarkable performance and generalizability of this module on various cell datasets significantly contribute to enhancing the overall effectiveness of the framework. In the Discussion section, a detailed experimental analysis will be provided to further validate its effectiveness and explore this aspect.

## Discussion

In this section, the segmentation performances of the proposed CMTT are evaluated under different situations. Different datasets and adaptation tasks are categorized. Corresponding experiments are conducted from easy to difficult to validate the performance of the CMTT.

###  

#### Evaluation metrics and experiment design

Several commonly utilized image segmentation metrics are introduced to evaluate the performance of various models. These metrics encompass both pixel-level and object-level measurements. In addition, this section outlines the fundamental experimental settings for the segmentation tasks.

The dice coefficient is widely used in segmentation applications and is defined by


(13) \begin{align*}& Dice(G, S) = \frac{2\vert G \cap S\vert}{\vert S \vert + \vert G \vert},\end{align*}

where $\vert G \vert $ and $\vert S \vert $ are pixels of the ground truth image and the associated segmented image, respectively. $\vert G \cap S \vert $ is the intersection of pixels from the two images. Similarly, IOU can be defined based on the Jaccard Similarity Index at the pixel level:


(14) \begin{align*}& IOU = \frac{\vert G \cap S \vert}{\vert G \cup S \vert}\end{align*}

We have also used the Aggregated Jaccard Index (AJI) [39] to assess the performance at the object level. The AJI is defined as an extension of the global Jaccard index as follows:


(15) \begin{align*}& AJI = \frac{\Sigma^{N}_{i=1} \vert G_{i} \cap S_{i} \vert}{\Sigma^{N}_{i=1} \vert G_{i} \cup S_{i} \vert + \Sigma_{i\in R}\vert S_{i}\vert},\end{align*}

where $S_{i}$ is the predicted object that maximizes the Jaccard index with ground truth object $G_{i}$, and $R$ is the set of segmented objects that do not match with the ground truth. Compared with IOU, AJI is even more stringent. The AJI reflects the ratio between the intersection area of the matching element and the segmentation results. Thus, any inaccurate segmentation (under-, over-, or mis-segmentation) will result in a reduction in AJI. IOU only compares whether pixels belonging to an object are recognized; it does not care if one object is recognized as two or two objects are recognized as one.

As per Supplementary File Section 1, adaptation performances can be evaluated sequentially, encompassing cross-cell-type and cross-microscopy-modality adaptation, large-scale dataset to small dataset adaptation, and sparse cell to dense cell adaptation.

###  

#### Benchmarking methods of segmentation

Popular cell segmentation models are considered as benchmarking methods in the computational experiment to verify the advantage of CMTT. Considered benchmarking methods include two groups, supervised and unsupervised. In the experiments, supervised methods are trained on the source domain and then tested on the target domain.

***Supervised methods*** U-Net [23] and Mask R-CNN [24], two widely used deep-learning-based image segmentation models, serve as benchmarking methods in this study. Additionally, significant research has been conducted on cell segmentation based on deep learning. Two notable works are selected as benchmarks. In [16], the authors combined a U-Net-based initial stage for cell-cluster or superpixel segmentation, with a secondary refinement stage using Faster R-CNN to detect small cell objects within connected clusters. A Mask R-CNN architecture with Shape-Aware Loss was adopted in [64] for cell segmentation in microscopy images.

All these supervised methods are trained on the source domain, and then fine-tuned on the target domain. It is important to note that, unlike these supervised methods, the upcoming unsupervised benchmarking methods and our CMTT do not require target domain annotations for training or testing. For those unsupervised methods, labeled data from the target domain are utilized solely to evaluate and compare model performance.

***Unsupervised methods*** As discussed before, traditional unsupervised cell segmentation methods are still widely used. Here, a band pass filter followed by thresholding, which was introduced in [11], is regarded as the traditional unsupervised benchmark.

Two unsupervised domain adaptation (UDA) or fully test-time adaptation methods have been implemented as benchmarks in this study. CyCADA, as introduced in [65], is a distinguished UDA technique specifically designed for image segmentation tasks. Moreover, in [66], a comprehensive fully test-time adaptation method was introduced tailored for image segmentation. The training process for the two methods should align with CMTT. First, the model is trained using training data from the source domain, followed by model selection using validation data from the source domain. Subsequently, unsupervised adaptation is performed within the target domain. Finally, the models are validated according to specific evaluation metrics on the target domain. To ensure comparability when integrating these domain adaptation approaches into cell segmentation tasks, it is imperative to maintain consistency in both the source and target domains with those used in CMTT. This consistent approach allows for an accurate comparison of model generalizability and adaptability across various domains and domain adaptation methods.

### Cross-cell-type and cross-microscopy-modality adaptation

Nowadays, many algorithms can achieve good results on the same type (same cell type, microscopy modality) of datasets after fine-tuning. Thus, we will not dwell on such domain adaptation. Instead, cross-cell-type and cross-microscopy-modality adaptations are first discussed. To facilitate the control of other variables, CTC datasets are chosen as an example.

Tables 9 and 10 demonstrate the performance of CMTT and the benchmarking methods for these adaptations. When the source domain is DIC-C2DH-HeLa and the target domain is PhC-C2DL-PSC, not only does the microscope modality change, but the cell type also changes. The additional two groups of experiments in Tables 9 and 10 involve changing either the microscope modality or the cell type while maintaining consistency in the other. Our CMTT demonstrates superior performance compared with benchmarks in scenarios involving changes in both microscope modality and cell type. Notably, the traditional thresholding method exhibits the weakest performance across various experiment types; yet, its efficacy remains relatively stable in the face of different adaptations of the source and target domains. Among the benchmarking methods, [66] performs better when both the microscope modality and cell type change, while Mask R-CNN performs better otherwise.

**Table 9:** Cross-cell-type adaptation comparison of the benchmarking methods and CMTT (PhC-C2DH-U373$\rightarrow $PhC-C2DL-PSC)

Methods		Dice$\uparrow $	AJI$\uparrow $	IOU$\uparrow $
Supervised	U-Net	0.893	0.815	0.852
	Mask-RCNN	0.923	0.859	0.893
	[16]	0.901	0.843	0.882
	[64]	0.899	0.832	0.866
Unsupervised	Traditional	0.768	0.702	0.701
	CyCADA	0.905	0.829	0.867
	[66]	0.912	0.831	0.860
CMTT		0.918	0.846	0.886

**Table 10:** Cross-microscopy-modality adaptation comparison of the benchmarking methods and CMTT

Methods		DIC-C2DH-HeLa$\rightarrow $Fluo-N2DL-HeLa			DIC-C2DH-HeLa$\rightarrow $PhC-C2DL-PSC		
		Dice$\uparrow $	AJI$\uparrow $	IOU$\uparrow $	Dice$\uparrow $	AJI$\uparrow $	IOU$\uparrow $
Supervised	U-Net	0.897	0.824	0.861	0.862	0.784	0.834
	Mask-RCNN	0.931	0.868	0.905	0.881	0.812	0.857
	[16]	0.913	0.851	0.872	0.869	0.804	0.824
	[64]	0.891	0.848	0.871	0.848	0.783	0.829
Unsupervised	Traditional	0.756	0.710	0.714	0.751	0.697	0.698
	CyCADA	0.911	0.841	0.872	0.901	0.826	0.864
	[66]	0.904	0.851	0.869	0.902	0.831	0.856
CMTT		0.928	0.877	0.890	0.914	0.842	0.880

Moreover, in scenarios where the source domain is DIC-C2DH-HeLa and the target domain is PhC-C2DL-PSC, the overall performance of different techniques tends to be poorer compared with situations where the target domain is Fluo-N2DL-HeLa, particularly noticeable with supervised benchmarking methods. This discrepancy highlights the varying effects that different microscope modalities and cell types can have on model segmentation performance. When only one of these variables changes, the adverse effect on model performance is relatively minor in datasets with high standardization levels like CTC datasets, where supervised segmentation techniques still exhibit certain advantages. However, when both variables change simultaneously, the resulting domain shift intensifies, leading to a more pronounced negative impact on model performance.

### Adaptation to small-scale datasets

Then, it turns to the more common practical problem: How can we adapt a model from well-annotated large-scale datasets to small-scale datasets, which may not match the large-scale dataset in all aspects of measuring conditions?

In Table 11, the adaptation from EVICAN to the small-scale public datasets in Section *Summary of Cell Datasets with Annotations* is taken as an example. From Table 11, the supervised methods perform significantly worse on small datasets, especially those with a large difference from the source domain. It can be noted that U-Net performs relatively well, probably because it is more adaptable to small datasets than Mask R-CNN. Also, as a one-stage method, U-Net has a lower number of parameters and is less difficult to train. There is no significant bias in the performance of the traditional unsupervised methods on large or small datasets, but the key lies in the accuracy of the parameter tuning and the suitability of the selected dataset for the method.

**Table 11:** Adaptation comparison of the benchmarking methods and CMTT to small-scale datasets (Dice Metric, numbering according to Fig. 2)

Methods		(C1)	(C2)	(C4)	(C5)	(C6)	(C7)	(C8)
Supervised	U-Net	0.783	0.765	0.752	0.786	0.783	0.863	0.802
	Mask-RCNN	0.743	0.759	0.733	0.747	0.744	0.829	0.786
	[16]	0.726	0.745	0.745	0.771	0.761	0.801	0.739
	[64]	0.729	0.711	0.739	0.764	0.763	0.811	0.743
Unsupervised	Traditional	0.768	0.742	0.721	0.766	0.762	0.834	0.797
	CyCADA	0.825	0.819	0.807	0.823	0.831	0.880	0.869
	[66]	0.829	0.809	0.813	0.821	0.839	0.874	0.879
CMTT		$\boldsymbol{0.898}$	$\boldsymbol{0.883}$	$\boldsymbol{0.890}$	$\boldsymbol{0.901}$	$\boldsymbol{0.911}$	$\boldsymbol{0.921}$	$\boldsymbol{0.931}$

**Figure 12:**
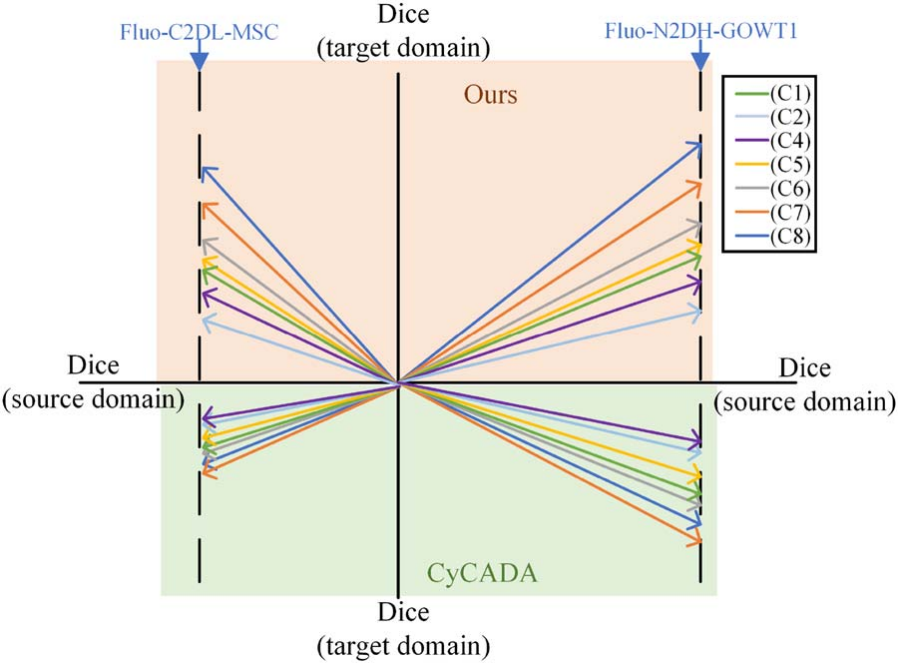
Comparison between adaptations from different source domains to the small-scale datasets.

In Fig. 12, the comparison between adaptations from two different source domains, Fluo-C2DL-MSC and Fluo-N2DH-GOWT1, to the small-scale public datasets is illustrated. The varying influence of the source domain on different domain adaptation methods can be illustrated. In particular, the performance of the source domain model in Fluo-C2DL-MSC is significantly inferior to that in Fluo-N2DH-GOWT1. CyCADA primarily enhances model performance by aligning the features of the source domain and the target domain during the training phase. Conversely, the fully test-time adaptation method relies on the source domain model to provide foundational performance and mainly improves adaptability in the target domain. As a result, the accuracy in the source domain has a more pronounced impact on CyCADA compared with our CMTT.

### Adaptation from sparse cells to dense cells

In cell segmentation, sparse cells are generally easier to identify than dense cells. Given that our dataset is significantly denser than the CTC dataset, in the final adaptation situation, the CTC dataset is treated as the source domain, while our dataset SWARM is treated as the target domain.

It can be seen in Table 12 that the traditional unsupervised method performs very poorly on dense cells; in fact, this serves as one major motivation of developing a fully test-time adaptation method in this work. The most widely used traditional unsupervised method is almost completely incapable of handling dense and overlapping cells. U-Net and Mask R-CNN also perform poorly because the target domain is too disparate from the source. The other UDA methods perform relatively well, but CMTT shows an absolute advantage.

**Table 12:** Adaptation comparison of the benchmarking methods and CMTT to SWARM

Methods		Dice$\uparrow $	AJI$\uparrow $	IOU$\uparrow $
Supervised	U-Net	0.463	0.465	0.401
	Mask-RCNN	0.392	0.379	0.366
	[16]	0.401	0.412	0.404
	[64]	0.412	0.407	0.393
Unsupervised	Traditional	0.268	0.282	0.291
	CyCADA	0.645	0.689	0.623
	[66]	0.624	0.665	0.579
CMTT		$\boldsymbol{0.758}$	$\boldsymbol{0.743}$	$\boldsymbol{0.694}$

**Figure 13:**
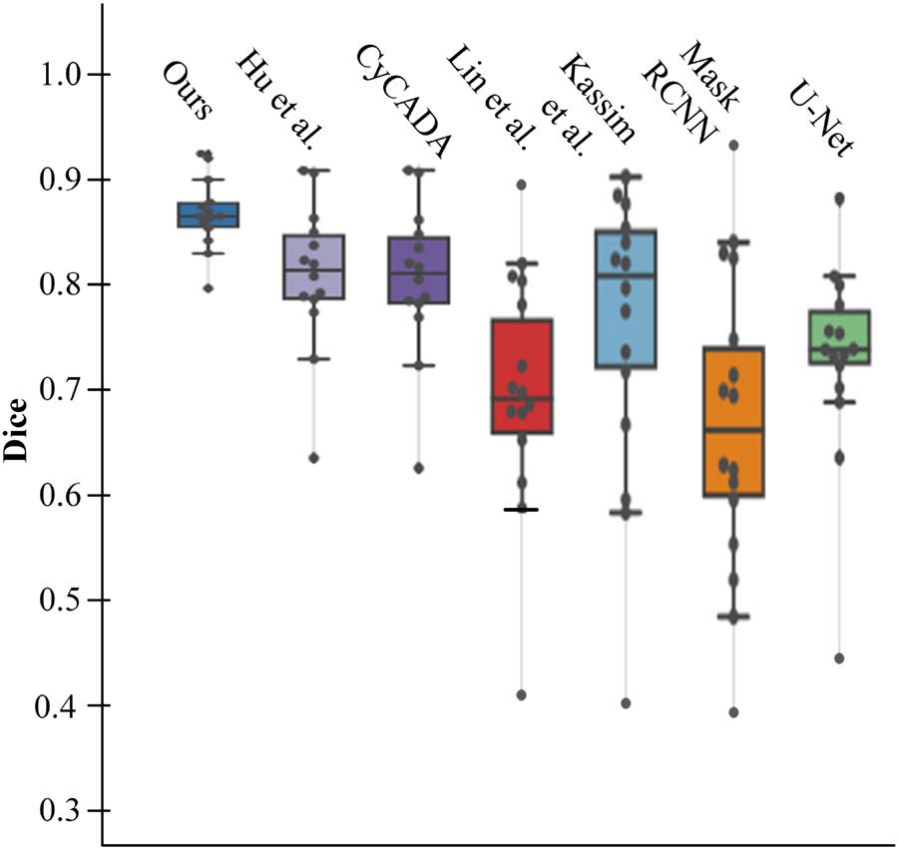
Boxplot of different frameworks on different adaptations.

The performance of all the aforementioned adaptations is summarized in Fig. 13. It is evident that our CMTT demonstrates favorable performance and stability across various source and target domain adaptations. In contrast, supervised methods exhibit lower overall stability, which can be attributed to their subpar performance on small-scale datasets and datasets lacking annotations, while performing well on larger datasets. However, the U-Net model exhibits relatively good stability, likely because of its simple and effective architecture, requiring fewer parameters and showing promising performance on small-scale datasets.

### The influence of different loss function terms in CMTT

This section elucidates the roles of different loss functions in CMTT through experiments. Two target domain datasets, DIC-C2DH-HeLa and Fluo-N2DH-GOWT1, are selected, while the source domain remained consistent as Fluo-N2DH-SIM+. The two target datasets are distinguished by unique features. In DIC-C2DH-HeLa, individual cell sizes dominate a larger portion of the total image area, with lower cell imaging density, indicating sparse cells. Conversely, in Fluo-N2DH-GOWT1, individual cells occupy a smaller portion of the overall image area, with higher cell imaging density, suggesting dense cells. The performance of CMTT on these datasets with different configurations of respective loss functions is outlined in Table 13. The corresponding loss function for Stage 2 (Patch Adaptation) is $\mathcal{L}_{CMC}$. The roles of Stage 2 and $\mathcal{L}_{CMC}$ are investigated by eliminating Stage 2 or replacing its loss function with Shannon entropy (with Stage 1 retained). For Stage 1, the corresponding loss terms are $\mathcal{L}_{s}$ and $\mathcal{L}_{bnm}$ in $\mathcal{L}_{E}$. The contributions of $\mathcal{L}_{s}$ and $\mathcal{L}_{bnm}$ are examined by varying $\alpha $ and removing $\mathcal{L}_{s}$ (with Stage 2 and $\mathcal{L}_{CMC}$ retained).

**Table 13:** Performance comparison of CMTT with different loss function settings

		DIC-C2DH-HeLa			Fluo-N2DH-GOWT1		
		Dice$\uparrow $	AJI$\uparrow $	IOU$\uparrow $	Dice$\uparrow $	AJI$\uparrow $	IOU$\uparrow $
No $\mathcal{L}_{s}$		0.872	0.870	0.867	0.859	0.801	0.845
With $\mathcal{L}_{s}$	$\alpha =0$	0.896	0.868	0.887	0.867	0.796	0.852
	$\alpha =0.2$	0.910	0.872	0.899	0.901	0.831	0.875
	$\alpha =0.5$	0.915	0.878	0.902	0.908	0.849	0.887
	$\alpha =1$	0.901	0.866	0.890	0.914	0.874	0.902
	$\alpha =2$	0.892	0.858	0.883	0.919	0.892	0.913
No Stage 2		0.875	0.832	0.861	0.877	0.854	0.880
Stage 2 With Shannon Loss		0.889	0.839	0.881	0.906	0.861	0.906

From Table 13, it is evident that when CMTT skips Stage 2, its performance degrades on both datasets across various metrics, with a more pronounced decline observed in Fluo-N2DH-GOWT1. This could be attributed to Stage 2 primarily focusing on refining the processing of dense cell. Substituting the loss of Stage 2 with Shannon entropy results in a noticeable performance decrease mainly in terms of AJI. AJI measures segmentation performance at the object level, whereas IOU and Dice only assess pixel-level performance, disregarding whether identified pixels belong to different objects. This suggests that $\mathcal{L}_{CMC}$, facilitated by contrastive learning, empowers CMTT to better differentiate between different cells, thereby achieving the intended design objective.

When CMTT goes through Stage 2, as indicated in Table 13, with the presence of $\mathcal{L}_{s}$, an increase in $\alpha $ leads to improved performance, particularly in AJI, in the Fluo-N2DH-GOWT1 dataset. This enhancement is attributed to $\mathcal{L}_{bnm}$ primarily enhancing recognition performance of the model near the decision boundary by reducing data point density. However, when $\mathcal{L}_{s}$ is removed, the model exhibits a decrease in performance in pixel-level metrics on both datasets, especially in DIC-C2DH-HeLa. This is because Shannon entropy provides a simple and efficient entropy solution for pixel identification across the entire image. Overemphasizing data near the decision boundary in datasets with low cell density potentially reduces the model efficiency since the overall image information is overlooked.

## Conclusion

In this study, we presented a central-metric fully test-time adaptive framework CMTT-JTracker for cell tracking tasks. This method maintained the concept of treating detection and ReID as a multi-task learning problem within the same network, following the outline in the JDE framework. Moreover, the CMTT paradigm was proposed to extract shared features for these two tasks, and the SA-ReID was designed to improve the performance of the ID embedding branch. The CMTT paradigm, based on test-time adaptation, allowed the model to capture feature representations at multiple scales without requiring additional training. The designed adaptation process and loss functions in this paradigm aimed to enrich and improve the accuracy of the pre-segmentation features extracted from different scales of the cell images. Additionally, the improved SA-ReID further consolidated these features and improved the learning of object-related representations. These improvements also aimed to reduce the competition between detection and ReID within the same network. To validate our framework, a comprehensive summary of commonly used public cell datasets was provided, along with the release of a new cell dataset containing segmentation annotations. Extensive experiments were conducted on these datasets, demonstrating that the CMTT-JTracker outperforms benchmarking methods in terms of overall tracking performance and biological relevance. Ablation studies confirmed the effectiveness of both CMTT and SA-ReID. Moreover, to validate the adaptation performance of CMTT as a domain adaptation method across different domains, it was tested on cross-cell-type and cross-microscopy-modality adaptation, as well as adaptation to smaller datasets and dense cell scenarios. The experiments indicated that the CMTT framework exhibited strong generalizability across different cell datasets. The gain in segmentation accuracy and efficiency for fully test-time unsupervised adaptation made it highly valuable for real-world scenarios.

Despite the promising performance of CMTT-JTracker, we acknowledge that there are still several limitations to the proposed CMTT and the experiment design. Firstly, although we designed many experiments to test the generalizability of CMTT-JTracker and the CMTT paradigm on different cellular datasets from different perspectives, data from a wider range of sources are still needed to validate the conclusion. Secondly, the small number of annotated images in our dataset, despite the large total number, did not guarantee whether the segmentation and tracking results are representative. This is also a common problem for cell image datasets. Moreover, the online association algorithm for cell tracking had limitations in comprehensively considering the impact of various factors on the tracking task. It fails to account for important information, such as the causal association of cells and events like cell division and cell death at different time points. This lack of comprehensive consideration might weaken the overall effectiveness of the tracking algorithm.

**Key Points**

- A novel central-metric fully test-time adaptive framework CMTT-JTracker is proposed for cell tracking tasks. Experimental results show that the proposed CMTT-JTracker achieves superior performance over other methods in terms of multiple evaluation standards and in various experiments.
- This work presents an innovative application of test-time adaptation to pre-segmentation in cell tracking, leading to the development of a central-metric two-stage fully test-time adaptation framework CMTT.
- CMTT enables incorporating microscopic features into the segmentation process, which has taken the microscopic structure into special consideration rather than solely rely on the contrast between the cell edge and the background.
- A spatial attention re-identification network (SA-ReID) is developed to enhance the learning of object-related representations and consolidate features across different scales. Ablation studies have been conducted to demonstrate the effectiveness of SA-ReID.
- A new well-annotated cell image dataset SWARM is provided. SWARM exhibits large image sizes and densely packed cells. Moreover, we provide a comprehensive summary of publicly available cell image datasets and nuclei image datasets with annotations.

## Supplementary Material

Supplementary_File_bbae591

## Data Availability

Code can be accessed at https://github.com/lynnwahh/CMTTJ. Dataset and the corresponding annotations can be accessed at https://osf.io/gb7zu/.

## References

[1] Meijering E . Neuron tracing in perspective. *Cytometry A* 2010;77:693–704. 10.1002/cyto.a.20895. 20583273

[2] Padfield D , Rittscher J, Roysam B. Coupled minimum-cost flow cell tracking for high-throughput quantitative analysis. *Med Image Anal* 2011;15:650–68. 10.1016/j.media.2010.07.006. 20864383

[3] Zimmer C , Labruyere E, Meas-Yedid V. et al. Segmentation and tracking of migrating cells in videomicroscopy with parametric active contours: A tool for cell-based drug testing. *IEEE Trans Med Imaging* 2002;21:1212–21. 10.1109/TMI.2002.806292. 12585703

[4] Li K , Miller ED, Chen M. et al. Cell population tracking and lineage construction with spatiotemporal context. *Med Image Anal* 2008;12:546–66. 10.1016/j.media.2008.06.001. 18656418 PMC2670445

[5] Marquet P , Rappaz B, Magistretti PJ. et al. Digital holographic microscopy: A noninvasive contrast imaging technique allowing quantitative visualization of living cells with subwavelength axial accuracy. *Opt Lett* 2005;30:468–70. 10.1364/OL.30.000468. 15789705

[6] Sanderson MJ , Smith I, Parker I. et al. Fluorescence microscopy. *Cold Spring Harb Protoc* 2014;2014:pdb.top071795. 10.1101/pdb.top071795. 25275114 PMC4711767

[7] Mertz J . Introduction to Optical Microscopy. Cambridge, UK: Cambridge University Press, 2019. 10.1017/9781108552660.

[8] Kheireddine S , Perumal AS, Smith ZJ. et al. Dual-phone illumination-imaging system for high resolution and large field of view multi-modal microscopy. *Lab Chip* 2019;19:825–36. 10.1039/C8LC00995C. 30698180

[9] Wang Z , Zheng L, Liu Y. et al. Towards real-time multi-object tracking. In: European conference on computer vision, pp. 107–22. Glasgow, UK: Springer, Andrea Vedaldi, 2020. 10.1007/978-3-030-58621-8_7.

[10] Liang C , Zhang Z, Zhou X. et al. Rethinking the competition between detection and reid in multiobject tracking. *IEEE Trans Image Process* 2022;31:3182–96. 10.1109/TIP.2022.3165376. 35412982

[11] Magnusson KEG , Jaldén J, Gilbert PM. et al. Global linking of cell tracks using the viterbi algorithm. *IEEE Trans Med Imaging* 2015;34:911–29. 10.1109/TMI.2014.2370951. 25415983 PMC4765504

[12] Nath SK , Palaniappan K, Bunyak F. Cell segmentation using coupled level sets and graph-vertex coloring. In: Medical Image Computing and Computer-Assisted Intervention–MICCAI 2006: 9th International Conference, Copenhagen, Denmark, October 1-6, 2006. Proceedings, Part I 9, pp. 101–8. Copenhagen, Denmark: Springer, Rasmus Larsen, 2006. 10.1007/11866565_13 PMC1995122 17354879

[13] Pereira PMM , Fonseca-Pinto R, Paiva RP. et al. Accurate segmentation of dermoscopic images based on local binary pattern clustering. In: 2019 42nd International Convention on Information and Communication Technology, Electronics and Microelectronics (MIPRO), pp. 314–9, Opatija, Croatia: IEEE, 2019.

[14] Hajdowska K , Student S, Borys D. Graph based method for cell segmentation and detection in live-cell fluorescence microscope imaging. *Biomed Signal Process Control* 2022;71:103071. 10.1016/j.bspc.2021.103071.

[15] Liu T , Zhang M, Javanmardi M. et al. Sshmt: Semi-supervised hierarchical merge tree for electron microscopy image segmentation. In: Computer Vision–ECCV 2016: 14th European Conference, Amsterdam, The Netherlands, October 11–14, 2016, Proceedings, Part I 14, pp. 144–59. Amsterdam, The Netherlands: Springer, Bastian Leibe, 2016.

[16] Kassim YM , Palaniappan K, Yang F. et al. Clustering-based dual deep learning architecture for detecting red blood cells in malaria diagnostic smears. *IEEE J Biomed Health Inform* 2021;25:1735–46. 10.1109/JBHI.2020.3034863. 33119516 PMC8127616

[17] Le Dinh , Lee S-H, Kwon S-G. et al. Cell nuclei segmentation in cryonuseg dataset using nested unet with efficientnet encoder. In: 2022 International Conference on Electronics, Information, and Communication (ICEIC), pp. 1–4, Shanghai,China: IEEE, S.P Joshi, 2022.

[18] Lin S , Norouzi N. An effective deep learning framework for cell segmentation in microscopy images. In: 2021 43rd Annual International Conference of the IEEE Engineering in Medicine & Biology Society (EMBC), pp. 3201–4, Virtual Conference: IEEE, Jim Xiuquan Ji, 2021. 10.1109/EMBC46164.2021.9629863 34891922

[19] Schwendy M , Unger RE, Parekh SH. Evican—A balanced dataset for algorithm development in cell and nucleus segmentation. *Bioinformatics* 2020;36:3863–70. 10.1093/bioinformatics/btaa225. 32239126 PMC7320615

[20] Merchant F , Castleman K. Microscope Image Processing. Amsterdam, The Netherlands: Elsevier, Fatima Merchant, 2022.

[21] Meijering E . Cell segmentation: 50 years down the road [life sciences]. *IEEE Signal Process Mag* 2012;29:140–5. 10.1109/MSP.2012.2204190.

[22] Ulman V , Maška M, Magnusson KEG. et al. An objective comparison of cell-tracking algorithms. *Nat Methods* 2017;14:1141–52. 10.1038/nmeth.4473. 29083403 PMC5777536

[23] Ronneberger O , Fischer P, Brox T. U-net: Convolutional networks for biomedical image segmentation. In: Medical Image Computing and Computer-Assisted Intervention–MICCAI 2015: 18th International Conference, Munich, Germany, October 5-9, 2015, Proceedings, Part III 18, pp. 234–41. Munich, Germany: Springer, Nassir Navab, 2015.

[24] He K , Gkioxari G, Dollár P. et al. Mask r-cnn. In: Proceedings of the IEEE international conference on computer vision, pp. 2961–9, Venice, Italy: IEEE, 2017.

[25] Payer C , Štern D, Neff T. et al. Instance segmentation and tracking with cosine embeddings and recurrent hourglass networks. In: International Conference on Medical Image Computing and Computer-Assisted Intervention, pp. 3–11. Granada, Spain: Springer, Alejandro F. Frangi, 2018. 10.1007/978-3-030-00934-2_1.

[26] Hayashida J , Nishimura K, Bise R. Mpm: Joint representation of motion and position map for cell tracking. In: Proceedings of the IEEE/CVF Conference on Computer Vision and Pattern Recognition, pp. 3823–32, Seattle, WA, USA: IEEE, 2020.

[27] Ker DFE , Eom S, Sanami S. et al. Phase contrast time-lapse microscopy datasets with automated and manual cell tracking annotations. *Scientific Data* 2018;5:180237. 10.1038/sdata.2018.237. 30422120 PMC6233481

[28] Magnusson KEG , Jaldén J. A batch algorithm using iterative application of the viterbi algorithm to track cells and construct cell lineages. In: 2012 9th IEEE International Symposium on Biomedical Imaging (ISBI), pp. 382–5, Barcelona, Spain: IEEE, 2012.

[29] Zhou Z , Wang F, Xi W. et al. Joint multi-frame detection and segmentation for multi-cell tracking. In: Image and Graphics: 10th International Conference, ICIG 2019, Beijing, China, August 23–25, 2019, Proceedings, Part II 10, pp. 435–46. Beijing, China: Springer,Yao Zhao, 2019.

[30] Dufour A , Thibeaux R, Labruyere E. et al. 3-d active meshes: Fast discrete deformable models for cell tracking in 3-d time-lapse microscopy. *IEEE Trans Image Process* 2010;20:1925–37. 10.1109/TIP.2010.2099125. 21193379

[31] Delgado-Gonzalo R , Chenouard N, Unser M. Fast parametric snakes for 3d microscopy. In: 2012 9th IEEE International Symposium on Biomedical Imaging (ISBI), pp. 852–5. Barcelona, Spain: Ieee, 2012.

[32] Amat F , Lemon W, Mossing DP. et al. Fast, accurate reconstruction of cell lineages from large-scale fluorescence microscopy data. *Nat Methods* 2014;11:951–8. 10.1038/nmeth.3036. 25042785

[33] Maška M , Daněk O, Garasa S. et al. Segmentation and shape tracking of whole fluorescent cells based on the chan–vese model. *IEEE Trans Med Imaging* 2013;32:995–1006. 10.1109/TMI.2013.2243463. 23372077

[34] CellTrackingChallenge . Cell Tracking Challenge 2020. http://celltrackingchallenge.net/.

[35] Kurmi VK , Subramanian VK, Namboodiri VP. Domain impression: A source data free domain adaptation method. In: Proceedings of the IEEE/CVF winter conference on applications of computer vision, pp. 615–25, Virtual Conference: IEEE, 2021.

[36] Ljosa V , Sokolnicki KL, Carpenter AE. Annotated high-throughput microscopy image sets for validation. *Nat Methods* 2012;9:637–7. 10.1038/nmeth.2083. 22743765 PMC3627348

[37] Zheng X , Wang Y, Wang G. et al. Fast and robust segmentation of white blood cell images by self-supervised learning. *Micron* 2018;107:55–71. 10.1016/j.micron.2018.01.010. 29425969

[38] Mahbod A , Schaefer G, Bancher B. et al. Cryonuseg: A dataset for nuclei instance segmentation of cryosectioned h&e-stained histological images. *Comput Biol Med* 2021;132:104349. 10.1016/j.compbiomed.2021.104349. 33774269

[39] Naylor P , Laé M, Reyal F. et al. Segmentation of nuclei in histopathology images by deep regression of the distance map. *IEEE Trans Med Imaging* 2019;38:448–59. 10.1109/TMI.2018.2865709. 30716022

[40] Kumar N , Verma R, Sharma S. et al. A dataset and a technique for generalized nuclear segmentation for computational pathology. *IEEE Trans Med Imaging* 2017;36:1550–60. 10.1109/TMI.2017.2677499. 28287963

[41] Janowczyk A , Madabhushi A. Deep learning for digital pathology image analysis: A comprehensive tutorial with selected use cases. *J Pathol Inf* 2016;7:29. 10.4103/2153-3539.186902. PMC4977982 27563488

[42] Caicedo JC , Goodman A, Karhohs KW. et al. Nucleus segmentation across imaging experiments: The 2018 data science bowl. *Nat Methods* 2019;16:1247–53. 10.1038/s41592-019-0612-7. 31636459 PMC6919559

[43] Koos K , Molnár J, Kelemen L. et al. Dic image reconstruction using an energy minimization framework to visualize optical path length distribution. *Sci Rep* 2016;6:1–9. 10.1038/srep30420. 27453091 PMC4958949

[44] Ren S , He K, Girshick R. et al. Faster r-cnn: Towards real-time object detection with region proposal networks. IEEE Transactions on Pattern Analysis and Machine Intelligence 2017, **39**:1137–1149. 10.1109/TPAMI.2016.2577031. 27295650

[45] Shannon CE . A mathematical theory of communication. *Bell Syst Tech J* 1948;27:379–423. 10.1002/j.1538-7305.1948.tb01338.x.

[46] Wang D , Shelhamer E, Liu S. et al. Tent: Fully test-time adaptation by entropy minimization In: International Conference on Learning Representations (ICLR) 2021, ICLR 2021 Spotlight. Vienna, Austria: ICLR, 2021. arXiv:2006.10726. 2021.

[47] Jin Y , Wang X, Long M. et al. Minimum class confusion for versatile domain adaptation. In: Computer Vision–ECCV 2020: 16th European Conference, Glasgow, UK, August 23–28, 2020, Proceedings, Part XXI 16, pp. 464–80. Glasgow, UK: Springer, Andrea Vedaldi, 2020.

[48] Ma A , Li J, Ke L. et al. Adversarial entropy optimization for unsupervised domain adaptation. *IEEE Trans Neural Networks Learn Syst* 2021;33:6263–74. 10.1109/TNNLS.2021.3073119. 33939616

[49] Cui S , Wang S, Zhuo J. et al. Towards discriminability and diversity: Batch nuclear-norm maximization under label insufficient situations. In: 2020 IEEE/CVF Conference on Computer Vision and Pattern Recognition (CVPR), pp. 3940–9. Seattle, WA, USA: IEEE, 2020.

[50] Papadopoulo T , Lourakis MLA. Estimating the Jacobian of the Singular Value Decomposition: Theory and Applications PhD thesis,. Sophia Antipolis Cedex, France: INRIA, 2000. 10.1007/3-540-45054-8_36.

[51] Sharma A , Kalluri T, Chandraker M. Instance level affinity-based transfer for unsupervised domain adaptation. In: 2021 IEEE/CVF Conference on Computer Vision and Pattern Recognition (CVPR), pp. 5357–67. Nashville, TN, USA: IEEE, 2021.

[52] Van der Maaten , Hinton G. Visualizing data using t-sne. *J Mach Learn Res* 2008;9:2579−2605.

[53] He K , Fan H, Wu Y. et al. Momentum contrast for unsupervised visual representation learning. In: Proceedings of the IEEE/CVF conference on computer vision and pattern recognition, pp. 9729–38, Seattle, WA, USA: IEEE, 2020.

[54] Wu Z , Xiong Y, Yu SX. et al. Unsupervised feature learning via non-parametric instance discrimination. In: Proceedings of the IEEE conference on computer vision and pattern recognition, pp. 3733–42, Salt Lake City, UT, USA: IEEE, 2018.

[55] Arora S , Khandeparkar H, Khodak M. et al. A theoretical analysis of contrastive unsupervised representation learning. In: Proceedings of the 36th International Conference on Machine Learning, ICML 2019, pp.5628–5637. Long Beach, California, USA: ICML, arXiv preprint arXiv:1902.09229. 2019.

[56] Li Y , Wang N, Shi J. et al. Adaptive batch normalization for practical domain adaptation. *Pattern Recognit* 2018;80:109–17. 10.1016/j.patcog.2018.03.005.

[57] Woo S , Park J, Lee J-Y. et al. Cbam: Convolutional block attention module. In: Proceedings of the European conference on computer vision (ECCV), pp. 3–19, Munich, Germany: Springer, Vittorio Ferrari, 2018.

[58] Redmon J , Farhadi A. Yolov3: An incremental improvement arXiv preprint arXiv:1804.02767. *Computer vision and pattern recognition.* Vol. **1804**, pp. 1–6. Berlin/Heidelberg, Germany: Springer, 2018.

[59] Lin T-Y , Goyal P, Girshick R. et al. Focal loss for dense object detection. In: Proceedings of the IEEE international conference on computer vision, pp. 2980–8, Venice, Italy: IEEE, 2017.

[60] Zheng Z , Wang P, Liu W. et al. Distance-iou loss: Faster and better learning for bounding box regression. In: Proceedings of the AAAI conference on artificial intelligence, Vol. 34, pp. 12993–3000, 2020. 10.1609/aaai.v34i07.6999. New York, NY, USA: AAAI, 2020.

[61] Zhou Z , Siddiquee MMR, Tajbakhsh N. et al. Unet++: A nested u-net architecture for medical image segmentation. In: Deep Learning in Medical Image Analysis and Multimodal Learning for Clinical Decision Support: 4th International Workshop, DLMIA 2018, and 8th International Workshop, ML-CDS 2018, Held in Conjunction with MICCAI 2018, Granada, Spain, September 20, 2018, Proceedings 4, pp. 3–11. Granada, Spain: Springer, Danail Stoyanov, 2018. 10.1007/978-3-030-00889-5_1 PMC7329239 32613207

[62] Bove A , Gradeci D, Fujita Y. et al. Local cellular neighborhood controls proliferation in cell competition. *Mol Biol Cell* 2017;28:3215–28. 10.1091/mbc.e17-06-0368. 28931601 PMC5687024

[63] Kasturi R , Goldgof D, Soundararajan P. et al. Framework for performance evaluation of face, text, and vehicle detection and tracking in video: Data, metrics, and protocol. *IEEE Trans Pattern Anal Mach Intell* 2009;31:319–36. 10.1109/TPAMI.2008.57. 19110496

[64] Lin S , Norouzi N. An effective deep learning framework for cell segmentation in microscopy images. In: 2021 43rd Annual International Conference of the IEEE Engineering in Medicine & Biology Society (EMBC), pp. 3201–4, Virtual Conference: IEEE, 2021. 10.1109/EMBC46164.2021.9629863 34891922

[65] Hoffman J , Tzeng E, Park T. et al. Cycada: Cycle-consistent adversarial domain adaptation. In: Proceedings of the 35th International Conference on Machine Learning; **80**:1989–1998. Stockholmsmässan, Stockholm Sweden: PMLR, arXiv:1711.03213. 2018.

[66] Minhao H . Fully test-time adaptation for image segmentation. In: de Bruijne, Cattin PC, Cotin S. et al. (eds.), *Medical Image Computing and Computer Assisted Intervention — MICCAI 2021, Lecture Notes in Computer Science*, pp. 251–60. Cham: Springer International Publishing, 2021.

