# Supplementary material for: CMTT-JTracker: a fully test-time adaptive framework serving automated cell lineage construction
Source: Brief Bioinform. 2024 Nov 17;25(6):bbae591. doi: 10.1093/bib/bbae591 (PMC11570544; doi:10.1093/bib/bbae591)
Supplement: Supplementary_File_bbae591 [file supplementary_file_bbae591.docx]

**Supplementary File of the Manuscript Entitled "CMTT-JTracker: A fully test-time adaptive framework serving automated cell lineage construction"**

Liuyin Chen,^1^ Sanyuan Fu^2^ and Zijun Zhang^1,∗^

^1^School of Data Science, City University of Hong Kong, Hong Kong SAR, China and ^2^ Department of Modern Physics and Hefei National Laboratory for Physical Sciences at Microscale, University of Science and Technology of China, Hefei, Anhui, China

^*^Corresponding author. [zijzhang@cityu.edu.hk](mailto:zijzhang@cityu.edu.hk)

**1. Experiment Design for The Discussion Section**

In the manuscript, the cell type and the microscopy modality of the different datasets are summarized. The simplest case should definitely be the domain adaptation between different datasets of the same cell type or microscopy modality, which is called inter-cell-type or inter-microscopy-modality adaptation in our manuscript. On the other hand, the adaptation between datasets from different cell types or microscopy modalities is called cross-cell-type or cross-microscopy-modality adaptation in the manuscript.

The problem of domain adaptation, and in particular cell segmentation, has an infinite number of possibilities for different combinations of domains. Therefore, in the experiment setup, instead of an exhaustive enumeration of the possibilities of adapting from each existing dataset to another, several different scenarios are presented in the order of an ascending difficulty. We have developed a comprehensive testing scheme for cell segmentation methods across various cell datasets. Experiments have been conducted to test them with the corresponding source and target datasets. In the *Discussion* Section in the manuscript, adaptation performances are tested in the order of considering the cross-cell-type and cross-microscopy-modality adaptation, the large-scale dataset to small dataset adaptation, and sparse cell to dense cell adaptation.

**2. The Influence of Different η of The Joint Loss on The Performance of CMTT-JTracker**

$$\begin{aligned} \mathcal{L}_{total}=\mathcal{L}_{det}+\eta\mathcal{L}_{id}\#\left( \text{}\text{ AUTONUMLGL \textbackslash* Arabic\textbackslash e }\text{} \right) \end{aligned}$$

From (1), it is evident that the joint loss comprises two components, the term $\mathcal{L}_{det}$ measuring the loss for the detection task and the term $\mathcal{L}_{id}$ measuring the loss for the ReID task. While these tasks can share features, they also exhibit a competing relationship. Achieving optimal tracking performance entails maintaining a relative equilibrium between these tasks by utilizing the joint loss. Therefore, $\eta$ in the joint loss for CMTT-JTracker is fine-tuned through preliminary experiments conducted on Fluo-N2DH-SIM+. The results are shown in Table 1. IDF_1_ [1] is introduced for the ReID task to specifically measure the accuracy of the model in maintaining the identity of tracked targets during the tracking process. Additionally, mAP [2] is incorporated to evaluate the performance of the detection task. Analysis from Table 1 reveals that, with a higher proportion of $\mathcal{L}_{det}$, the model demonstrates enhanced performance in terms of mAP. Conversely, an increase in $\mathcal{L}_{id}$ proportion leads to improved IDF_1_ performance. The overall tracking performance of the model is assessed using MOTA, where setting η to 0.05 establishes a relative balance between mAP and IDF_1_, resulting in the best tracking performance of CMTT-JTracker.

Table 1. **The performance of the CMTT-JTracker for Fluo-N2DH-SIM+ dataset with different η values**

| η | MOTA↑ | IDF_1_↑ | mAP(%)↑ |
| --- | --- | --- | --- |
| 0.001 | 0.811 | 0.843 | 90.4 |
| 0.01 | 0.852 | 0.876 | 89.5 |
| 0.02 | 0.864 | 0.883 | 89.0 |
| 0.05 | 0.872 | 0.896 | 88.2 |
| 0.1 | 0.848 | 0.909 | 87.6 |

**Reference**

[1] Ristani, E., Solera, F., Zou, R., Cucchiara, R., & Tomasi, C. (2016, October). Performance measures and a data set for multi-target, multi-camera tracking. In European conference on computer vision (pp. 17-35). Cham: Springer International Publishing.

[2] Everingham, M., Van Gool, L., Williams, C. K., Winn, J., & Zisserman, A. (2010). The pascal visual object classes (voc) challenge. International journal of computer vision, 88, 303-338.
